# Challenges in dengue research: A computational perspective

**DOI:** 10.1111/eva.12554

**Published:** 2017-11-05

**Authors:** José Lourenço, Warren Tennant, Nuno R. Faria, Andrew Walker, Sunetra Gupta, Mario Recker

**Affiliations:** ^1^ Department of Zoology University of Oxford Oxford UK; ^2^ Centre for Mathematics and the Environment University of Exeter Penryn UK

**Keywords:** computation, dengue, epidemiology, evolution, models

## Abstract

The dengue virus is now the most widespread arbovirus affecting human populations, causing significant economic and social impact in South America and South‐East Asia. Increasing urbanization and globalization, coupled with insufficient resources for control, misguided policies or lack of political will, and expansion of its mosquito vectors are some of the reasons why interventions have so far failed to curb this major public health problem. Computational approaches have elucidated on dengue's population dynamics with the aim to provide not only a better understanding of the evolution and epidemiology of the virus but also robust intervention strategies. It is clear, however, that these have been insufficient to address key aspects of dengue's biology, many of which will play a crucial role for the success of future control programmes, including vaccination. Within a multiscale perspective on this biological system, with the aim of linking evolutionary, ecological and epidemiological thinking, as well as to expand on classic modelling assumptions, we here propose, discuss and exemplify a few major computational avenues—real‐time computational analysis of genetic data, phylodynamic modelling frameworks, within‐host model frameworks and GPU‐accelerated computing. We argue that these emerging approaches should offer valuable research opportunities over the coming years, as previously applied and demonstrated in the context of other pathogens.

## 1 BACKGROUND

### 1.1 The dengue virus

Dengue virus (DENV) is a single‐stranded, positive‐sense RNA virus with a population comprised of four antigenically distinct groups, or serotypes (DENV1–4), that present similar population dynamics and are the cause of clinically indistinguishable illnesses in humans (Gubler, 1998; Halstead, 2007; Kyle & Harris, 2008). Classification based on genomic organization, homology and antigenic cross‐reactivity place DENV into the *Flavivirus* genus of the viral family *Flaviviridae* (Calisher, Karabatsos, Dalrymple, Shope, & Porterfield, 1989). More than 70 flaviviruses have so far been identified (Kuno, Chang, Tsuchiya, Karabatsos, & Cropp, 1998), many of which are important human pathogens, including the hepatitis C virus (HCV), Japanese encephalitis virus (JEV), West Nile virus (WNV), yellow fever virus (YFV) and the recently emerged Zika virus (ZIKV). Among the ones that are vector‐born (all except HCV), DENV is responsible for the highest human morbidity and mortality, with children carrying the highest burden of disease (Anders et al., 2011; Jain & Chaturvedi, 2010; Murray, Quam, & Wilder‐Smith, 2013).

### 1.2 Dengue evolution

Observations were made in the late 1970s of dengue‐like viruses and respective animal reservoirs in Asia and West Africa (Aiken & Leigh, 1978). Since then, a wide number of sylvatic strains (i.e., of animal origin) have been isolated and reported to belong to each of the lineages of human‐bearing serotypes, proposing a zoonotic origin for the pathogen (Holmes & Twiddy, 2003). Introduction of each serotype into the human population was extrapolated to have taken place at different time points, with DENV2's most recent common ancestor being the oldest, estimated to be approximately 1,000 years old (Twiddy, Holmes, & Rambaut, 2003; Weaver & Vasilakis, 2009), roughly corresponding to some of the first reports of dengue‐like illnesses (Vasilakis & Weaver, 2008; Tang et al., 2010; Weaver & Vasilakis, 2009). The genetic distance between currently circulating serotypes is likely a by‐product of allopatric evolution due to ecological partitioning of ancestral sylvatic strains (Holmes & Twiddy, 2003; Weaver & Vasilakis, 2009). This hypothesis is sustained not only by DENV's current population structure, but also by studies that report sylvatic and endemic (i.e., human bearing) strains to exhibit similar evolutionary rates and (cell‐) infection potential (Twiddy et al., 2002; Vasilakis et al., 2007).

Within each serotype, nucleic acid sequencing techniques have identified genetic subgroups or lineages, commonly denominated as dengue genotypes (Tang et al., 2010; Weaver & Vasilakis, 2009). Notably, the overall amino acid sequence similarity among genotypes is generally above 90% (Holmes & Twiddy, 2003; Leitmeyer et al., 1999), whereas between serotypes, it can range between 60% and 80% (Irie, Mohan, Sasaguri, Putnak, & Padmanabhan, 1989; Rothman, 2011). Although RNA viruses generally present rapid evolution and accumulation of diversity occurring at a similar time‐scale as their host's ecological dynamics (Grenfell, Pybus, Gog, Wood, & Daly, 2004), studies have demonstrated that strong purifying selection dominates DENV's recent evolution (Bennett, Holmes, Chirivella, Rodriguez, & Beltran, 2003; Holmes, 2003; Holmes & Twiddy, 2003; Weaver & Vasilakis, 2009; Zhang, Mammen, Chinnawirotpisan, Klungthong, & Rodpradit, 2005). A prevailing hypothesis is that the virus has evolved to replicate efficiently in both the vertebrate and arthropod hosts (Filomatori, Carballeda, Villordo, Aguirre, & Pallarés, 2017), expressing a compromise genome in which most amino acid changes are expected to be deleterious and selectively removed from the population (Holmes, 2003; Holmes & Twiddy, 2003; Weaver & Vasilakis, 2009). The lack of recombination, supported by rare reports on naturally occurring recombinants (Waman, Kolekar, Ramtirthkar, Kale, & Kulkarni‐Kale, 2016; Weaver & Vasilakis, 2009), is further believed to limit the fixation and emergence of single, positively selected mutations due to clonal interference (Grenfell et al., 2004; Lourenço & Recker, 2010; Miralles, Gerrish, Moya, & Elena, 1999).

### 1.3 Dengue transmission cycle

Dengue virus is transmitted to humans by the bite of infectious mosquitoes. Two mosquito species have been identified as the main disease vectors: *Aedes aegypti* and *Ae. albopictus*. Intercontinental changes in the second half of the 20th century, in particular the post‐World War II boom in urbanization and globalization, have facilitated the introduction and establishment of dengue's two principal mosquito vectors into many major urban and peri‐urban settings and resulted in the worldwide establishment of endemic transmission cycles (i.e., between humans; Vasilakis, & Weaver, 2008; Murray et al., 2013; Weaver & Vasilakis, 2009). *Ae. aegypti* is particularly domesticated and often uses artificial water containers found within and around human habitats as breeding sites. Adult mosquitoes are optional haematophages, but females require blood for egg production and show strong preference for human blood meals (Scott, Morrison, Lorenz, Clark, & Strickman, 2000).

In contrast, the transmission cycle of sylvatic viruses takes place in forest environments of South‐East Asia and West Africa between nonhuman primates and arboreal *Aedes* species. Rural populations living in close proximity to forested or plantation areas are periodically exposed to such variants (Holmes & Twiddy, 2003; Vasilakis, & Weaver, 2008; Weaver & Vasilakis, 2009). Secondary transmission is rarely reported, which may be a combined result of limited human‐to‐vector transmission potential, underreporting due to low pathogenicity, weak surveillance and lack of peridomestic vectors (*Ae. aegypti* and *Ae. albopictus*).

### 1.4 Dengue epidemiology

The epidemiological picture of dengue across the world is variable. While hyperendemicity is classically associated with South‐East Asia, the 21st century has witnessed the endemic establishment of the virus in South and Central America (Brathwaite Dick, San Martín, Montoya, del Diego, & Zambrano, 2012; San Martín, Brathwaite, Zambrano, Solórzano, & Bouckenooghe, 2010). Dengue has also recently emerged in the United States, with repeated but so far self‐contained outbreaks in Florida and Texas (Guzman, Halstead, Artsob, Buchy, & Farrar, 2010; Radke, Gregory, Kintziger, Sauber‐Schatz, & Hunsperger, 2012; Thomas, Santiago, Abeyta, Hinojosa, & Torres‐Velasquez, 2016). In Europe, outbreaks in Italy, France, Greece and the Madeira island (Portugal) have been reported in recent years, but with no observations of endemic circulation (Cavrini, Gaibani, Pierro, Rossini, & Landini, 2009; Gautret, Botelho‐Nevers, Charrel, & Parola, 2010; Gjenero‐Margan, Aleraj, Krajcar, Lesnikar, & Klobučar, 2011; Lourenço & Recker, 2014).

Poor surveillance, no official reporting to the WHO (Jaenisch, Junghanss, Wills, Brady, & Eckerle, 2014) and the high prevalence of other diseases makes the situation in Africa more uncertain. Recently, outbreaks of dengue or dengue‐like illness were reported in dry countries of Eastern Africa and South West Asia, such as Saudi Arabia (Aziz, Al‐Shami, Mahyoub, Hatabbi, & Ahmad, 2014), Yemen (Bin Ghouth, Amarasinghe, & Letson, 2012), Sudan (Seidahmed, Hassan, Soghaier, Siam, & Ahmed, 2012) and Madagascar (Schwarz, Girmann, Randriamampionona, Bialonski, & Maus, 2012). In 2004 and 2009, epidemics occurred in the isolated populations of Cape Verde (Franco, Di Caro, Carletti, Vapalahti, & Renaudat, 2010), Mauritius islands (Issack, 2010) and Reunion island (Larrieu, Dehecq, Balleydier, Jaffar, & Michault, 2012), and a major epidemic in Pakistan took place in 2013 (Kraemer, Perkins, Cummings, Zakar, & Hay, 2015; Khan, Khan, & Amin, 2016). Together with the widespread presence of *Ae. aegypti* and *Ae. albopictus*, this would indicate that the burden of dengue in Africa is likely to be worse than previously acknowledged (Guzman et al., 2010; Jaenisch et al., 2014; Kyle & Harris, 2008).

**Figure 1:**
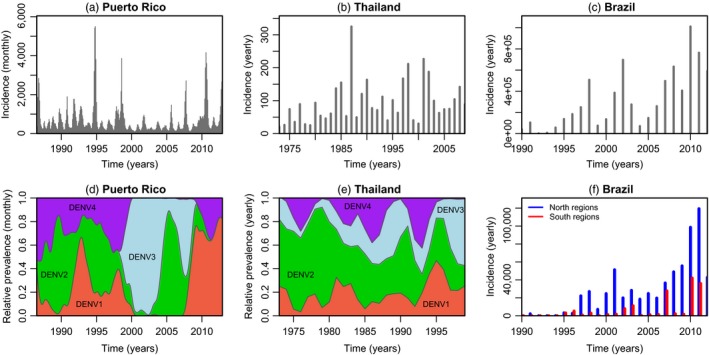
Epidemiological time series of reported dengue cases for three different countries. (a) Total monthly incidence for Puerto Rico. (b) Annual dengue haemorrhagic fever incidence per 100,000 individuals in Thailand. (c) Total annual incidence for Brazil. (d) Monthly serotype, relative prevalence for Puerto Rico. (e) Yearly serotype, relative prevalence for Thailand. (f) Yearly absolute incidence for the north and south of Brazil (see *Appendix: Data Description* for details)

Driven by seasonal variation in vector abundances, incidence patterns are usually characterized by annual outbreaks (Figure 1a; Cuong, Hien, Duong, Phong, & Cam, 2011; Lourenço & Recker, 2013; Nisalak, Endy, Nimmannitya, Kalayanarooj, & Thisayakorn, 2003). In addition, multi‐annual epidemics with a 2‐ to 5‐year period have also been reported in highly endemic settings (Figure 1a,b; Bennett, Drummond, Kapan, Suchard, & Muñoz‐Jordán, 2010; Cummings, Irizarry, Huang, Endy, & Nisalak, 2004; Cuong et al., 2011; Lourenço & Recker, 2013; Nisalak et al., 2003; Thai, Cazelles, Nguyen, Vo, & Boni, 2010). In contrast, regions where transmission has been historically low or where dengue serotypes were only recently introduced are more likely to show wave‐like, increasing patterns of incidence (Figure 1c,f). Spatial heterogeneities are also common in reported data, highlighting that the success of dengue viruses can be dictated by numerous demographic and ecological determinants (Figure 1f; Adams & Kapan, 2009; Beebe, Cooper, Mottram, & Sweeney, 2009; Campbell, Haldeman, Lehnig, Munayco, & Halsey, 2015; Chew, Woon, Amin, Adnan, & Abdul Wahab, 2016; Cummings et al., 2004; Lourenço & Recker, 2010; Lourenço & Recker, 2013).

Where all four serotypes cocirculate endemically, relative prevalence levels of each serotype are characterized by oscillatory behaviour with periods of 8–10 years (Figure 1d,e; Adams, Holmes, Zhang, Mammen, & Nimmannitya, 2006; Bennett et al., 2010; Lourenço & Recker, 2013; Nisalak et al., 2003). Whereas incidence dynamics are thought to be driven by a complex interplay of local factors, from the abundance of susceptible humans, climate and viral traits, to the density and distribution of both the human and the vector populations (Johansson, Dominici, & Glass, 2009; Morrison, Minnick, Rocha, Forshey, & Stoddard, 2010; Nagao, Svasti, Tawatsin, & Thavara, 2008; Tsuzuki, Vu, Higa, Nguyen, & Takagi, 2009; Williams, Bader, Kearney, Ritchie, & Russell, 2010), there is lack of evidence that these factors directly influence the qualitative properties of serotype cyclical behaviour, which seems to be a universal feature of dengue epidemiology.

### 1.5 Human infections, immunity and disease

Primary infections in humans with any of the four serotypes are generally mild with the majority of cases believed to be asymptomatic (Kyle & Harris, 2008; Nisalak et al., 2003). Infections usually result in a short‐lived, febrile illness, which is commonly referred to as *dengue fever* (DF) and is characterized by arthralgia (joint pain), myalgia (muscle pain), low‐grade fever and retro‐orbital headaches (Balmaseda, Hammond, Tellez, Imhoff, & Rodriguez, 2006; Burke, Nisalak, Johnson, & Scott, 1988; Wichmann, Yoon, Vong, Limkittikul, & Gibbons, 2011). In some cases, this may progress to more severe and life‐threatening forms of disease—*dengue haemorrhagic fever* (DHF) or *dengue shock syndrome* (DSS)—with manifestations of circulatory failure, vascular permeability and haemorrhagic symptoms (Gubler, 1998; Halstead, 2007).

Flaviviruses are known to induce the phenomenon of *original antigenic sin* (OAS) (Francis, 1960), in which B‐cell clones sequentially challenged by viruses of the same family initially respond by synthesizing antibodies with higher affinity to a previous infecting virus (Halstead, Rojanasuphot, & Sangkawibha, 1983). This creates a cascade of regulatory changes that facilitates expression and synthesis of pro‐inflammatory cytokines and induces a positive feedback that increases the rate of intracellular infection, leading to higher viraemia levels and worse clinical outcomes (Beltramello, Williams, Simmons, Macagno, & Simonelli, 2010; Fernandez‐Garcia, Mazzon, Jacobs, & Amara, 2009; Green, Vaughn, Kalayanarooj, Nimmannitya, & Suntayakorn, 1999; Ubol & Halstead, 2010; Whitehorn & Simmons, 2011). In dengue infections, this cascade is commonly referred to as *antibody‐dependent enhancement* (ADE) and is mainly associated with heterologous DENV challenges (Balsitis, Williams, Lachica, Flores, & Kyle, 2010; Halstead, Mahalingam, Marovich, Ubol, & Mosser, 2010; Wrammert, Onlamoon, Akondy, Perng, & Polsrila, 2012) or the presence of maternal antibodies in infants (Chau, Hieu, Anders, Wolbers, & Lien, 2009).

Generally, cellular immune responses (T cell) to DENV are seen to cover most of the viral proteins (Duangchinda, Dejnirattisai, Vasanawathana, Limpitikul, & Tangthawornchaikul, 2010; Rothman, 2011), but with a particular dominant signal for NS3 (Duangchinda et al., 2010), which represents only ≈20% of DENV amino acid coding sequence (Rothman, 2011). T‐cell clones can be serotype cross‐reactive, sero‐specific or even strain‐specific (i.e., within serotype; Bashyam, Green, & Rothman, 2006; Mangada, Endy, Nisalak, Chunsuttiwat, & Vaughn, 2002; Rothman, 2011; Sierra, García, Pérez, Morier, & Rodríguez, 2002; Ubol & Halstead, 2010). The cross‐reactivity is not surprising, however, given the ≈70% amino acid identity between DENV1 and DENV4 (Rothman, 2011). OAS in T‐cell responses, including inflammation and cytokine‐induced alteration of vascular permeability (Rothman, 2011), have also been described in secondary infections (Mongkolsapaya, Dejnirattisai, Xiao‐ning, Vasanawathana, & Tangthawornchaikul, 2003), with consequences for pathogenicity (Bashyam et al., 2006; Duangchinda et al., 2010; Imrie, Meeks, Gurary, Sukhbataar, & Kitsutani, 2007; Mangada et al., 2002; Stephens, Klaythong, Sirikong, Vaughn, & Green, 2002).

Hence, available evidence from both humoral and cellular immunity suggests that the clinical outcome of DENV infection is finely tuned to an individual's immune response, in particular to the degree of OAS and associated inflammatory levels. Factors that may influence the regulation of an individual's response, in particular for secondary challenges, might include the following: host genetics, viral strain, sequence of infecting serotypes, age, co‐infection with other pathogens, etc. However, there is a major gap in our current knowledge on reliable, immunological markers for clinical outcomes, and discerning between such factors is often difficult (Rothman, 2011), although, generally, the most significant risk factor for severe disease is secondary infection with an heterologous serotype as a primer for OAS (Burke et al., 1988; Guzman & Kouri, 2008; Halstead et al., 2010; Kyle & Harris, 2008; Sangkawibha, Rojanasuphot, Ahandrik, Viriyapongse, & Jatanasen, 1984; Thein, Aung, Shwe, Aye, & Zaw, 1997; Rothman & Ennis, 1999; Halstead & O'Rourke, 1977; Dejnirattisai, Jumnainsong, Onsirisakul, Fitton, & Vasanawathana, 2010).

Recovery is thought to result in lifelong protection against the infecting serotype (homologous immunity) (but see (Forshey, Reiner, Olkowski, Morrison, & Espinoza, 2016; Waggoner, Balmaseda, Gresh, Sahoo, & Montoya, 2016) for possible incomplete protection). A period of 6–36 months of temporary cross‐protective immunity to all serotypes has also been suggested after recovery (see e.g., Sabin, 1952; Reich, Shrestha, King, Rohani, & Lessler, 2013), although it is still uncertain whether this form of immunity solely protects against clinical disease or completely prevents infection (Bhoomiboonchoo, Nisalak, Chansatiporn, Yoon, & Kalayanarooj, 2015; Anderson et al., 2014; Kochel, Watts, Halstead, Hayes, & Espinoza, 2002; Kochel, Watts, Gozalo, Ewing, & Porter, 2005; Olkowski, Forshey, Morrison, Rocha, & Vilcarromero, 2013). Upon recovery from a secondary, heterologous infection, humans are believed to be (clinically) protected against further challenges. Due to the rarity of third and fourth infections in clinical data and the difficulties in assessing an individual's infection history because of high antibody cross‐reactivity in serological assays (Halstead, 2007; Nisalak et al., 2003; Olkowski et al., 2013; Gibbons, Kalanarooj, Jarman, Nisalak, & Vaughn, 2007), it is not yet clear to what degree secondary infection prevents re‐infections or simply renders subsequent infections, even by previously un‐experienced serotypes, clinically silent.

### 1.6 Immune interactions and computational approaches

**Figure 2:**
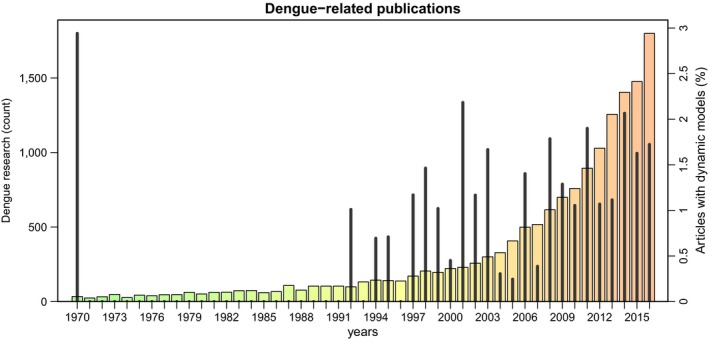
Dengue publication over the last five decades. Total number of dengue articles per year (bars) and the percentage of those with a computational focus (spikes). Between 1970 and 2016, a total of 15,267 dengue articles were published, including 190 modelling studies (see Appendix for details)

Mathematical and computational models are recognized as an important tool in infectious disease epidemiology and have been applied to dengue since the mid‐20th century. Hand‐in‐hand with the increase in the global distribution and burden of dengue, the number of modelling studies has also gone up (Figure 2). Topics have ranged widely, from estimations of dengue's reproductive number to the impact of climate and space on disease transmission and spread, to the evaluation and assessment of disease control strategies. Dengue's complex epidemiological dynamics, with multi‐annual outbreaks and sequential replacement of dominant serotypes, has also attracted a fair share of the attention of the modelling community, with many studies concentrating on immune interactions between dengue serotypes, either through ADE and/or by means of cross‐immunity. In fact, the incorporation of immune interaction has become ubiquitous to most dengue models and hence deserves a little more detail here.

It is broadly recognized that immune competition can act to desynchronize antigenically diverse pathogens, such as dengue, malaria (*Plasmodium falciparum*) or the influenza A virus, and cause nonlinear or chaotic oscillations not too dissimilar to the observed epidemiological patterns of dengue. For dengue, competition between serotypes can be driven by ADE of secondary, heterologous infections or through (temporary or permanent) cross‐immunity. Both types of interactions are assumed to affect the transmissibility of subsequent infections, leading to frequency‐dependent selection that has been demonstrated to generate oscillatory behaviour and further facilitate serotype persistence (Schwartz, Shaw, Cummings, Billings, & McCrary, 2005; Ferguson, Anderson, & Gupta, 1999; Cummings, Schwartz, Billings, Shaw, & Burke, 2005; Aguiar, Kooi, & Stollenwerk, 2008; Wearing & Rohani, 2006; Adams & Boots, 2006; Hu, Thoens, Bianco, Edlund, & Davis, 2013).

With regard to ADE, initial criticism concerning the level of enhancement required for serotype desynchronization could be countered by also assuming enhancement in the susceptibility to secondary infections (see e.g., (Recker, Blyuss, Simmons, Hien, & Wills, 2009)). Importantly, however, empirical evidence supporting a universal increase in the transmission potential of secondary infections is still lacking. That is, while the ADE phenomenon has been shown both in in vitro and in vivo studies (Halstead et al., 2010; Dejnirattisai et al., 2010; Halstead, 2011), its actual onsequences for disease transmission and the proportion of hosts contributing to ADE‐induced phenotypes at any given time are largely unknown.

Cross‐immunity, where an infection by one serotype negatively affects the fitness of subsequently infecting serotypes, has equally been put forward in modelling studies to explain dengue's complex epidemiology (Adams et al., 2006; Reich et al., 2013; Aguiar et al., 2008; Wearing & Rohani, 2006). Recent studies on prospective cohorts have found that shorter time periods between sequential infections are associated with some degree of clinical protection (Anderson et al., 2014; Montoya, Gresh, Mercado, Williams, & Vargas, 2013). However, apart from a single human study conducted in the 1950s (Sabin, 1952), experimental support for both the nature and duration of cross‐immunity is still lacking. Consequently, there is no broad consensus about the protective efficacy of the observed cross‐reactivity between dengue serotypes and whether cross‐reactive responses prevent infection or solely act to alleviate clinical outcomes. Model assumptions have therefore diverged significantly with regard to the duration and the type of protection following a primary dengue infection.

It is worth noting that some of the controversies surrounding serotype immune interactions and their effects on the epidemiological dynamics of dengue are partially consequences of the actual modelling frameworks themselves. That is, deterministic models based on mass‐action principles, which comprise the majority of mathematical models found in the published literature, require an interaction term in order to desynchronize the serotypes and to exhibit dengue's characteristic multi‐annual incidence patterns. Stochastic, and in particular individual‐based, models do not rely on this assumption to generate epidemiological time series with such properties. The contrasting dynamic behaviour is driven by the amplification of stochastic effects at the individual‐level (demographic stochasticity), which keeps each serotype/strain in a transient regime rather than approaching the expected deterministic equilibrium even in the absence of explicit desynchronizing factors (Lourenço & Recker, 2013; Alonso, McKane, & Pascual, 2007). Crucially, understanding the epidemiological consequences of dengue immune interactions goes beyond the theoretical argument and has recently been shown to be of major importance for dengue vaccination impact studies based on mathematical models (Lourenço & Recker, 2016; Flasche et al., 2016).

## 2 CHALLENGES AND OPPORTUNITIES IN COMPUTATIONAL RESEARCH

The zoonotic origin, separate endemic and sylvatic transmission cycles, immunological serotype interactions and the vast diversity of dengue genotypes provide a valuable background for integrative evolutionary approaches in the context of dengue. However, the predominant focus on single biological scales (e.g., host‐host transmission) has so far limited our capacity to link important population genetic and ecological observations with the pathogen's epidemiological behaviour within a single framework. In sharp contrast to other major viral pathogens, such as the influenza A virus or the human immunodeficiency virus (HIV), evolutionary frameworks do not feature prominently in dengue's modelling literature, with the field of phylogenetics so far dominating evolutionary approaches.

In general, the continuing geographical and epidemic expansion of dengue's genetic variants and mosquito vectors, and the failure to prevent the introduction and establishment of sustained transmission across the globe, call for a renewed focus and advancement in computational approaches. In the context of dynamic modelling, the literature has been dominated by frameworks based on ordinary differential equations (ODE), which are tractable and computationally inexpensive. However, such approaches often take key assumptions unquestioningly, such as homogeneous mixing, competition factors to desynchronize antigenic types, or continuity and determinism, which can hamper our understanding of the major determinants of dengue's evolution, epidemiology and control. Within a multiscale perspective on this biological system, and with the aim of linking evolutionary, ecological and epidemiological aspects as well as to expand on classic modelling assumptions, we here propose, discuss and exemplify a few major computational avenues, which we hope should offer valuable research opportunities over the coming years: real‐time computational analysis of genetic data, phylodynamic modelling frameworks, within‐host model frameworks and GPU‐accelerated computing.

### 2.1 Real‐time collection and computational analysis of genetic data

Like the yellow fever virus (Auguste, Lemey, Pybus, Suchard, & Salas, 2010), DENV was probably first introduced in the Americas through infected hosts and vectors during the slave trade (Weaver & Vasilakis, 2009; Vasilakis, & Weaver, 2008). At present, the distribution of DENV serotypes results from a combination of strong population structure and gene flow across geographical regions. South‐East Asia harbours the greatest DENV genetic diversity, and it is from there that viral lineages seem to seed epidemics in the Americas and elsewhere (Holmes, 2008). Several studies suggest that every 7–10 years, DENV genotypes become extinct and the replacement by new viral lineages leads to period fluctuations in genetic diversity (Klungthong, Zhang, Mammen, Ubol, & Holmes, 2004; Thu, Lowry, Jiang, Hlaing, & Holmes, 2005; Nunes, Palacios, Faria, Sousa, & Pantoja, 2014). Each of these epidemics is fuelled by a combination of local herd susceptibility (Salje, Lessler, Endy, Curriero, & Gibbons, 2012), seasonal abundance of the *Ae. aegypti* mosquito (Kraemer, Sinka, Duda, Mylne, & Shearer, 2015) and an increasingly well‐connected mobility network that facilitates the movement of infected hosts and vectors (Nunes et al., 2014; Wesolowski, Qureshi, Boni, Sundsøy, & Johansson, 2015).

Dengue virus incidence is often underestimated and novel surveillance, tools need to be applied to improve current estimates and reduce misclassification (Silva, Rodrigues, Paploski, Kikuti, & Kasper, 2016). The most commonly used dengue diagnostic tests are based on antibody detection and often show cross‐reactivity with other flaviviruses, including West Nile virus, Japanese encephalitis virus and yellow fever virus. Classification based on clinical symptoms alone is complicated by the circulation of pathogens that cause similar clinical symptoms to DENV infection, such as the chikungunya virus (CHIKV) and ZIKV, and may thus hamper accurate descriptions of DENV dynamics (Faria, Lourenço, Marques de Cerqueira, Maia de Lima, & Carlos Junior Alcantara, 2016; Roth, Mercier, Lepers, Hoy, & Duituturaga, 2014).

One of the ultimate challenges in infectious disease control is to predict and halt the course of an epidemic following its emergence. However, insufficient surveillance can obfuscate predictions of virus dynamics across local, regional and global scales. For instance, diagnostic tools and reporting are unevenly supported across locations with risk for transmission. This is particularly the case for central Africa, for which little information is known about the expansion of arboviruses (Jaenisch et al., 2014), although molecular detection from returning travellers has revealed active undetected transmission of DENV and CHIKV in this region (Parreira, Centeno‐Lima, Lopes, Portugal‐Calisto, & Constantino, 2014) and has identified it as the source of the CHIKV East‐Central‐South‐African genotype in the American continents (Nunes, Faria, de Vasconcelos, Golding, & Kraemer, 2015). It is also the case that representative data are essential to quantify the burden of transmission and to predict dynamic behaviour and impact of interventions (Jaenisch et al., 2014). However, even in regions with access to diagnosis and surveillance, it is rarely the case that reported case data on arboviruses are representative (Silva et al., 2016; Lourenco, Maia de Lima, Faria, Walker, & Kraemer, 2017). A major example for which both of these issues are particularly challenging is the Amazon region, that has been suggested to play a stepping stone role in the introduction of DENV lineages from the Caribbean into Brazil (Temporão, Penna, Carmo, Coelho, & Azevedo, 2011; Nunes, Faria, Vasconcelos, de Almeida Medeiros, & de Lima, 2012), and may have also been involved in the emergence and maintenance of ZIKV (Faria, Quick, Claro, Thézé, & de Jesus, 2017).

With the ongoing democratization of pathogen sequencing (Faria, Sabino, Nunes, Alcantara, & Loman, 2016), it is hoped that the generation of genetic data will soon become standard in public health and clinical practise (Houldcroft, Beale, & Breuer, 2017). Public databases are now becoming populated with thousands of partial and complete genome sequences. Sequencing during epidemics is even becoming an increasing trend, which is important for our understanding on how particular genotypes associated with increased virulence may be generated and eventually fixate (OhAinle, Balmaseda, Macalalad, Tellez, & Zody, 2011). Moreover, genetic data collected over the course of months, years and decades allow for a better understanding of the dynamics of DENV lineages at the population level, offering key insights into demographic, ecological and genetic factors which are difficult, if not impossible, to track with clinical records alone.

With the *real‐time genomic epidemiology revolution* (Gardy, Loman, & Rambaut, 2015), new and complementing insights (to clinical reported case data) are expected to provide unique information about the macro‐ and microscale process shaping viral dynamics. Statistical models that take into account shared ancestry allow testing for the contribution of demographic factors associated with virus persistence and spread (Faria, Suchard, Rambaut, Streicker, & Lemey, 2013; Lemey, Rambaut, Bedford, Faria, & Bielejec, 2014). Using such approaches, air travel has been shown to play a pivotal role in the spread of vectors (Tatem, Hay, & Rogers, 2006) and DENV (Nunes et al., 2014). Mobility may also play a significant role in the spread of arboviruses at more local scales; for example, time to travel has been shown to be strongly associated with incidence within distinct neighbourhoods of the same city during an arboviral outbreak (Faria, Lourenço et al., 2016). As real‐time collection of genetic data becomes more frequent and available, increased time and spatial resolution will allow future phylogenetic and modelling studies to further evaluate the relative contribution of short‐ and long‐distance mobility.

It may seem that these recently established approaches and real‐time sequencing efforts are more appropriate for emerging viruses, such as ZIKV or the Ebola virus (Faria et al., 2017; Woolhouse, Rambaut, & Kellam, 2015) and that opportunities have been missed in the context of DENV, given its generalized geographical spread and endemicity. However, major gaps in our knowledge still exist in the context of DENV, sustained by the insufficiency of clinical reported case counts. In particular, how genotypes manage to persist in face of significant herd immunity, on the relative role of urban versus rural spread routes, off‐season persistence and on the invasion and emergence of pathogenic lineages for which the displacement of the Asian/American by the Asian‐1 type in South‐East Asia is a good example (Lourenço & Recker, 2010; Hang, Holmes, Duong, Nguyen, & Tran, 2010). Hence, while the endemic state of DENV may render efforts of real‐time sequencing less appealing, we argue that the enumerated knowledge gaps offer major research opportunities. It is also the case that retrospective analysis of archived clinical samples, such as blood banks, could contribute massively. However, real‐time sequencing not only offers the same opportunities as retrospective approaches, as it also renders timely interventions possible with significant public health impact, for instance in face of emerging virulent genotypes or introduction of serotypes which may increase the number of heterologous challenges.

### 2.2 Phylodynamic modelling frameworks

The genetic diversity of RNA viruses is shaped in direct response to changes in host and vector demographic and ecological factors, such as population size, structure or movement. This provides measurable connections between host population dynamics, viral evolution and transmission. In recent years, genetic, immunological and epidemiological data have been successfully used to investigate such connections, unifying key observations across biological scales within a single framework known as phylodynamics (Grenfell et al., 2004; Volz, Koelle, & Bedford, 2013). However, while some studies have addressed dengue's epidemiology in the context of a phylodynamic framework (Rasmussen, Boni, & Koelle, 2014; Ke, Aaskov, Holmes, & Lloyd‐Smith, 2013), integrative modelling approaches in which transmission models are used to simulate both the epidemiology and molecular evolution of the virus have not yet been applied to dengue.

Dengue virus is a surprisingly conserved RNA virus (Choudhury, Lott, Banu, Cheng, & Teo, 2015; Lequime, Fontaine, Ar Gouilh, Moltini‐Conclois, & Lambrechts, 2016; Thai, Henn, Zody, Tricou, & Nguyet, 2012; Amarilla, de Almeida, Jorge, Alfonso, & de Castro‐Jorge, 2009; Holmes, 2003). Even though its serotypes present seemingly competitive dynamics, similar to the behaviour of antigenic strains of other fast evolving pathogens, observations of positive selection are rare (Tang et al., 2010; Filomatori et al., 2017; Waman et al., 2016; Hang et al., 2010; Choudhury et al., 2015; Martin, Chirivella, Co, Santiago, & Gubler, 2016; Sim, Aw, Wilm, Teoh, & Hue, 2015). Dengue transmission models have offered insight, but have so far failed to reach consensus on the possible biological drivers of the observed dynamic behaviour. For other pathogens, such as influenza A, phylodynamic transmission models have allowed to filter out a wide range of hypotheses regarding potential drivers (e.g., Bedford, Suchard, Lemey, Dudas, & Gregory, 2014; Minayev & Ferguson, 2009), as model validation becomes stricter and requires both epidemiological and evolutionary trajectories to match observed data patterns.

Here, we argue that phylodynamic models could offer critical insight into currently open questions on dengue's evolution and epidemiology, for example by assessing what assumptions about serotype immune interactions are compatible with empirical population genetic and phylogenetic data, similar to approaches applied to influenza A. This approach is exemplified in Box I, where we extended a previously published, individual‐based dengue transmission model (Lourenço & Recker, 2013) to include explicit molecular evolution. Two scenarios are compared: one in which the dynamics and evolutionary histories are purely driven by demographic and transmission processes without immune interactions (Box I, a–d), and one where we considered temporary, serotype‐transcending immunity following recovery from infection (Box I, e–f). With this approach, the phylogenetic output would be particularly informative as cross‐immunity results in more imbalanced phylogenetic trees, akin to a ladder‐like topology that is often reported for other pathogens, such as influenza A (Bedford et al., 2014; Bedford, Cobey, & Pascual, 2011) or within‐host HIV (Shankarappa, 1999), where cross‐immunity is known to play a crucial role.

Box I **Phylodynamic modelling**

1 The explicit modelling of molecular evolution allows for an exploration of dengue's drivers for key observations of phylogenetics and population genetics, such as tree imbalance, Tajima D and measures of viral diversity. Here, in order to illustrate such potential, we expand an individual‐based framework to include the transmission and evolution of individual virions. We make a number of simplifying assumptions: one virion is modelled per host; the transmission chain of a single serotype is used to define the discrete birth–death process of virions; mutation occurs over the birth–death process, resulting in a multichotomous, evolution tree; a fixed molecular clock is used for the mutational input, which follows an infinite‐sites model with no recombination; all mutations are assumed to be neutral; a burn‐in period of 25 years for transmission (ending at *t = *0 in figures), followed by 10 years for molecular evolution (*t = *10), is considered before model output is recorded; a single founder virus is used (*t = *0); population genetic sampling is uniform in time‐ and frequency‐dependent; phylogenetic tree is a small sample (<1%) of the evolution tree.

(a, b, c, d) Model output without considering serotype‐immune interactions. (e, f) Model output with 9 months of transcending cross‐immunity. A particular serotype (DENV1, orange) is chosen for the population genetics and phylogenetic output. Effective population size is considered to be the total number of virions of the chosen serotype. Some output variables are normalized between 0 and 1 for visualization purposes. (a and e) Dynamics of the four serotypes. (b) Tajima D (magenta) and effective population size (orange). (c) Number of segregating sites (light blue), sequence pairwise diversity (dark blue) and effective population size (orange). (d and f) Phylogenetic three with tips coloured according to accumulated number of mutations (tips are sampled after the 10 year burn‐in).

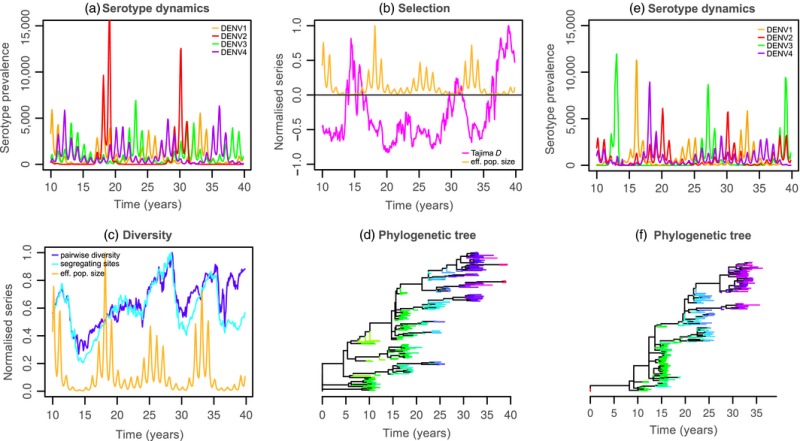

These figures present the dynamic potential of an individual‐based model with explicit molecular evolution. In figures a–d, the complete model output shows how demographic stochasticity and the mechanics of transmission alone can dictate the intrinsic behaviour of viral diversity and phylogenetic shape. In contrast, figures e and f show how such signatures (e.g. phylogentic shape) can be significantly altered when changing model assumptions—in this particular case, the addition of a 9‐month period of transcending, cross‐protective immunity.

Given seasonal epidemic troughs in endemic regions, possible mechanisms for viral persistence are still open topics of discussion, in particular as phylogenetic studies have shown that the lack of case reports can be followed by resurgence of previously circulating genetic variants (Bennett et al., 2003, 2010; Carrington, Foster, Pybus, Bennett, & Holmes, 2005). Persistence may be achieved by a combination of temporary circulation in the vector population (i.e., vertical transmission) (Bosio, Thomas, Grimstad, & Rai, 1992; Adams & Boots, 2010), an unknown reservoir (Weaver & Vasilakis, 2009; Vasilakis, Cardosa, Hanley, Holmes, & Weaver, 2012), silent transmission (Kyle & Harris, 2008; Morrison et al., 2010; Poblap, Nitatpattana, Chaimarin, Barbazan, & Chauvancy, 2006), or may simply be a consequence of reintroduction from nearby regions following local extinction. The particular hypothesis of continuous silent transmission is critical for public health, while the possibility of exogenous circulation has severe implications for control. Box I shows that viral diversity can be addressed in phylodynamic models, which is seen to respond both to seasonal troughs and to natural fluctuations in serotype prevalence. Diversity can be expected to behave differently, for example depending on whether exogenous reservoirs are considered or not. Phylodynamic models could thus be used to explore hypotheses on mechanisms of (local) viral persistence in ways not possible with current transmission models and may therefore change our current understanding of the persistence of dengue viruses.

In a broader evolutionary perspective, drivers of key observations on the molecular evolution of dengue could also be studied within these frameworks. For instance, strong purifying selection is consistently reported in sequence data, for which it is generally accepted that the two‐host life cycle imposes strong evolutionary constraints. Box I (b) shows, however, that even without explicit constraints on molecular evolution (e.g., deleterious mutations), negative Tajima D, a proxy for purifying selection, is self‐emergent. As such, demographic and transmission processes appear sufficient to maintain the viral population as under purifying selection. Sensitivity experiments could therefore be used to test which processes seem to dictate these population genetic signatures; for example, how does host mixing in terms of mobility or patch organization alter such observations?

There are also reports of sporadic episodes of positive selection and fixation of particular lineages (Tang et al., 2010; Filomatori et al., 2017; Waman et al., 2016; Hang et al., 2010; Choudhury et al., 2015; Martin et al., 2016), and it is generally believed that most events of lineage replacement go unnoticed, as the majority of endemic regions rely on syndromic surveillance, and as these events are not expected to result in significant changes in disease incidence (Lourenço & Recker, 2010). While there are rare examples of well‐described lineage shifts in the literature (OhAinle et al., 2011; Bennett, Holmes, Chirivella, Rodriguez, & Beltran, 2006), not much is known about the epidemiological and ecological contexts in which emergence of fitter lineages is favoured. Our previous work has suggested that stochastic effects and clonal interference are major determinants of the emergence of fitter, potentially more virulent variants (Lourenço & Recker, 2010). With these more detailed individual‐based model frameworks, the strength of such determinants could be tested with higher resolution, discerning, for instance, between stochastic pressures dictated by population structure, host mixing or spatial heterogeneity in herd immunity (Lourenço & Recker, 2013).

Disparate levels of within‐ and between‐host genetic diversity are a common feature of DENV viruses, and complementary hypotheses exist for these observations (Choudhury et al., 2015; Lequime et al., 2016; Thai et al., 2012). By allowing parameterization of mutational input and quantification on how population‐level events modulate within‐host genetic diversity, phylodynamic models could help make predictions on the relative contribution of different mechanisms. In particular, transmission bottlenecks could be implemented and their effect tested in the background of cross‐immunity and clonal interference (Lourenço & Recker, 2010).

There are concerns related to viral escape variants following intervention programmes based on vaccination and/or Wolbachia‐infected mosquitoes. While for Wolbachia‐based control strategies, it can be argued that these concerns are so far theoretical (Bull & Turelli, 2013), escape in a postvaccination era has been reported for a wide range of pathogens (Read, Baigent, Powers, Kgosana, & Blackwell, 2015; Pérez‐Sautu, Costafreda, Caylà, Tortajada, & Lite, 2011). The explicit generation, emergence and spread of escape mutants could be modelled within phylodynamic frameworks such as the one presented in Box I, given that individual mutations are already tracked. Vaccination, as demonstrated in previous work, can also be easily included (Lourenço & Recker, 2016; Flasche et al., 2016). Parameterization on the efficiency of viral escape to vaccine‐induced immunity would allow for sensitivity experiments and projections on the public health impact of such escape variants. Furthermore, demographic and ecological heterogeneities could also be researched, such as population structure, in order to evaluate under which conditions such variants could more easily emerge and devise appropriate intervention schemes that could stop their fixation.

Finally, as described in the previous section, real‐time collection and analysis of genetic data will soon offer vast amounts of high‐resolution data on population genetic measurements and phylogenetics, both in time and space. This will fundamentally change our perspective and the number of empirical observations that can be used to validate model frameworks. The use of more complex modelling approaches, such as individual‐based models, including atypical dimensions such as detailed spatial scales (Box II) or molecular evolution (Box I), will not only be a valuable path of research, but could indeed become necessary approaches to be able to match the rich variety of observed data patterns across biological scales.

Box II **Sensitivity analysis using GPU acceleration**

1 The increased computational power due to GPU parallelization permits a deeper exploration into the epidemiological effects of different community structures, vector and host heterogeneities, environmental heterogeneities or human movement patterns. Here, in order to illustrate the use of GPU‐accelerated agent‐based modelling, we investigate how the connectivity between subpopulations, as a proxy for human movement/major commuting patterns, affects the epidemiological dynamics of dengue within a spatial meta‐population model. In this framework, the population (human and mosquitoes) is divided into subpopulations and arranged spatially into a network of communities, connected by major trade and/or commuting routes. Dengue transmission events are dispersed through the network predominantly via local connections between communities as well as occasional but random transmission events between distant (i.e., not directly connected) subpopulations. The global connectivity pattern can then be quantified by means of the network's *degree bias*, which, in its simplest form, can be interpreted as the number of nodes (i.e., communities) that are only connected to one other node.

Plots a–b show two examples of networks with either a low or a high degree bias, respectively. As can be seen in the corresponding time series of serotype‐specific incidence, the way that communities are connected to form a meta‐population has a strong effect on the epidemiological dynamics, especially with respect to the interannual variability and serotype periodicity. This can be further illustrated by means of a sensitivity analysis, where we gradually increased the degree bias and recorded the model's relative change in four characteristic epidemiological and serotype‐specific measures (plots c–d). In this specific example, where we considered a population size of 5 million humans and up to 6 million mosquitoes, we gained a speed‐up of around 320 (using a Nvidia GTX 1070 graphics card). In other words, running the same analysis, which took just over 9 hr to complete, would have taken about 4 months on a single core CPU and still many weeks using multithreaded CPU computing on a high‐end PC. This clearly illustrates how GPU‐based computing may facilitate the use of bigger and more complex models for addressing some of the outstanding dengue epidemiological and evolutionary questions discussed in this work.

(a, b) The degree of connectivity between subpopulations has a strong effect on the epidemiological dynamics of dengue. (c, d) Model sensitivity analysis showing the relative change in epidemiological and serotype‐specific characteristics depending on the network's degree bias. Each point is the result of 50 model runs over a 100‐year period, with error bars depicting the standard error.

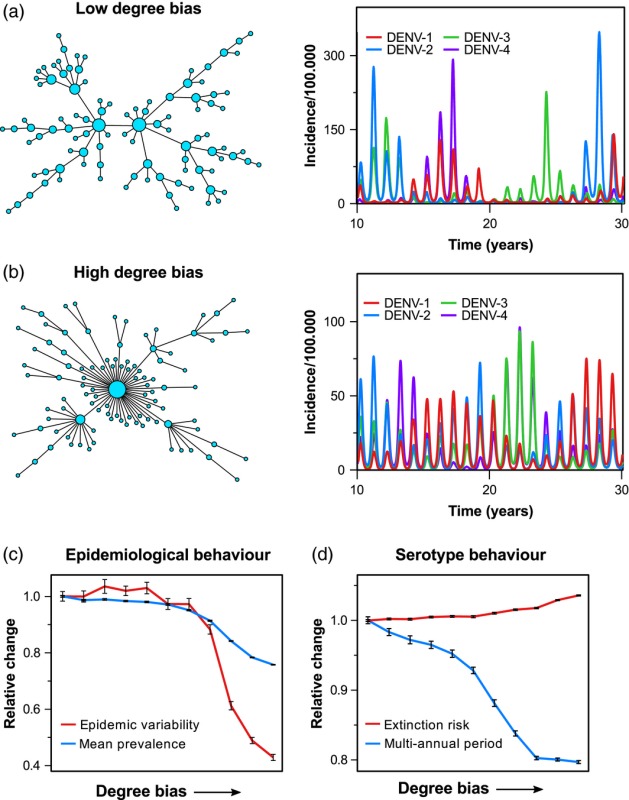

### 2.3 Within‐host models

The vast majority of dengue models has focused on viral dynamics at the population‐level. Given the importance that the within‐host processes (e.g., ADE and cross‐immunity) play for both viral transmission and disease pathology, it is thus surprising that just over a handful of studies have so far investigated viral dynamics at the within‐host level (Nuraini, Tasman, Soewono, & Sidarto, 2009; Ansari & Hesaaraki, 2012; Clapham, Tricou, N. V., Simmons, & Ferguson, 2014; Ben‐Shachar & Koelle, 2014; Gujarati & Ambika, 2014; Nikin‐Beers & Ciupe, 2015; Clapham, Quyen, Kien, Dorigatti, & Simmons, 2016; Ben‐Shachar, Schmidler, & Koelle, 2016). This could, however, be explained by the little understanding we currently have about the interactions between the virus, its target cells and the host immune responses that eventually clear infection, which may also be directly responsible for clinical outcome.

The target cell population for DENV replication is still not well characterized in vivo, although in vitro studies strongly suggest that monocytes, dendritic, endothelial and epithelial cells are among the preferential targets of infection (Fernandez‐Garcia et al., 2009; Ubol & Halstead, 2010; Dejnirattisai et al., 2010; Katzelnick, Coloma, & Harris, 2017). Similarly, it has been demonstrated that both innate and adaptive immune responses play a role during infection, but it is not well understood how these can mechanistically and synergistically achieve viral clearance.

There are two main known mechanisms of antibody action for viral clearance in dengue infection—antibodies can indirectly act to clear infected cells via antibody‐dependent cell‐mediated cytotoxicity (ADCC), or directly by neutralization. A data‐driven study by Clapham et al. (2016) focused on the distinction between these two mechanisms and found that either could explain the data (viral and antibody titres) equally well. Two other studies have focused on the general mechanics of humoral responses (B cell and free antibody) using different modelling frameworks (Gujarati & Ambika, 2014; Nikin‐Beers & Ciupe, 2015). While one study concluded that enhancing antibodies (ADE) were key in secondary infections (Gujarati & Ambika, 2014), the other study suggested instead a direct decrease of overall heterologous viral clearance, whereby cross‐reactive antibodies render virions unavailable for further binding and subsequent clearance by ADCC (Nikin‐Beers & Ciupe, 2015). In another study, the question about the role of the adaptive and innate immune responses in disease severity was addressed (Ben‐Shachar & Koelle, 2014; Ben‐Shachar et al., 2016). It was shown that characteristic features of primary infection could be replicated solely by innate immune responses (NK cells). In contrast, features of secondary infection, including ones related to clinical outcome, required greater infectivity rates (ADE) together with T cell‐mediated clearance of infected cells in a process arguably similar to well‐described cytokine storms (Rothman, 2011; Katzelnick et al., 2017). In line with previous results by Clapham et al. (2014), it was proposed that the observed variations in viral load could largely be explained by assuming patient‐specific incubation periods.

Generally and similarly to the aforementioned discrepancy between model assumptions related to immune interactions at the population level, within‐host approaches have been able to replicate important and ubiquitous data patterns, but, either due to formalism or data restrictions, have been limited in establishing a consensus framework. In some cases, the models and the data sets differed, making it hard to assess whether contrasting conclusions are data‐ or model‐driven. For instance, some models propose a key role of innate immunity in clearance of primary infection (Ben‐Shachar & Koelle, 2014; Ben‐Shachar et al., 2016), while others propose that either humoral or cell‐mediated clearance is required (Nuraini et al., 2009; Ansari & Hesaaraki, 2012; Clapham et al., 2014; Gujarati & Ambika, 2014; Nikin‐Beers & Ciupe, 2015; Clapham et al., 2016). Equally, asymmetries between serotypes in both cell infectivity and timing of peak viraemia versus symptomatic manifestations were reported in one study (Ben‐Shachar et al., 2016) but not in another (Clapham et al., 2014).

A current major problem is the lack of fine‐scaled data that captures the early, prepeak infection dynamics. This issue arises from the self‐limiting nature of dengue infections, fast viral clearance rates and the absence of antiviral therapies against dengue, which effectively prevent human infection studies. In fact, antiviral therapy has been devalued due to the fact that the timing of clinical symptoms often overlaps with peak viral load (Clapham et al., 2014); that is, most patients will seek treatment at a point when the virus is already being cleared by the immune system. However, in another example of contrasting observations, if there are indeed significant differences between the serotypes, as suggested by Ben‐Shachar et al. (Ben‐Shachar et al., 2016), then there might be a question whether sero‐specific strategies could be beneficial, for which modelling studies could contribute useful answers.

Another promising avenue is in the context of vaccination. Recently, the first dengue vaccine was licensed (Dengvaxia^®^) and many others are in advanced trial stages (Schmitz, Roehrig, Barrett, & Hombach, 2011; Thomas & Endy, 2011; Schwartz, Halloran, Durbin, & Longini, 2015). For a good number of these, data will be made available on population‐level efficacy but also on within‐host markers and dynamic measurements of infection and immune response (e.g., Dorigatti, Aguas, Donnelly, Guy, & Coudeville, 2015). Such data can be used to inform new modelling frameworks to explain vaccine action (e.g., infection versus transmission blocking), which may have considerable impact on licensing and future distribution. We note, however, that is yet unknown whether fine‐scale kinetic data will be available, which is essential to parameterize models (Magnus, 2013) and to add insight into current knowledge gaps on the fast kinetics of the virus, in particular during the initial phase of infection. Finally, and similar to between‐host models, within‐host models of dengue have so far only considered a single ecological scale. With the advent of models with better computational implementation (see next section), both the between‐host and within‐host dynamics could be integrated in unified, multiscale frameworks. These could be used to investigate how assumptions about viral dynamics and immune kinetics, together with individual‐level variations and transmission mechanics, would influence epidemiological and evolutionary observations at the population level (Box I).

### 2.4 GPU modelling frameworks

Many traditional approaches in theoretical epidemiology of dengue and other infectious diseases rely on mass‐action principles whereby the rate at which a disease spreads through a population is directly proportional to either the number or the proportion of infected and susceptible individuals within that population. Under these assumptions, every individual has the same probability of getting infected, contributes equally to disease transmission and recovers at the same rate as everyone else. Their low computational footprint and analytical tractability make these models an attractive choice for investigating population‐level dynamical behaviours, especially when homogeneity in time and space can safely be assumed. In many cases, this cannot be guaranteed, however, and individual variations, arising through the stochastic nature of infection events, for example, or through ecological and demographic heterogeneities, cannot be captured by these models and require a different approach altogether.

**Figure 3:**
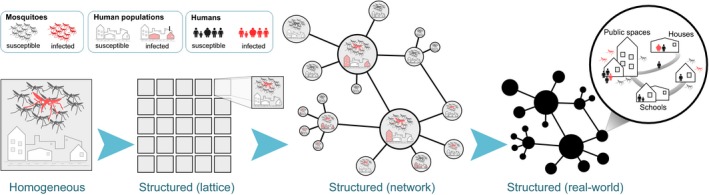
Increasing model complexity demands higher computational power. Model detail can be added by dividing a well‐mixed population into separate subpopulations, arranged in a regular spatial grid or by means of complex networks to represent geographical distribution of villages, towns and cities, with edges corresponding to major human movement patterns. Depending on data availability, more spatial and demographic detail can be added by considering individual households, places of work or schools. However, the computational demands increase significantly with more detailed information to keep track of, making the model very setting‐specific and impractical for sensitivity analyses and model fitting exercises

Agent or individual‐based models offer a natural and intuitive way to account for these variations by keeping track of individual, probabilistic infection events in both humans and mosquito vectors and have been implemented to various degrees of realism and model complexity. As an example, spatial details can be included by dividing the population into smaller subpopulations arranged in a rectangular grid (see Figure 3). This approach has already highlighted how the spatial segregation of individuals, causing stochastic, local extinction and re‐invasion, can explain much of dengue's observed epidemiology (Lourenço & Recker, 2013). Further detail may be introduced by adding more realistic spatial arrangements by means of complex networks with nodes representing villages or cities and edges representing their connecting trade or commuting routes, for example. Subpopulations can further be divided to take into account individual households, work places or schools, as well as the human movement patterns between them (see, e.g., (Chao, Halstead, Halloran, & Longini, 2012; Karl, Halder, Kelso, Ritchie, & Milne, 2014; Perkins, Garcia, Paz‐Soldán, Stoddard, & Reiner, 2014)). In fact, the explicit description of each individual (human and vector), together with their ecological and socio‐demographic interactions, allows a near limitless level of spatio‐temporal detail to be incorporated into these models.

The inclusion of (every) minute detail comes at the cost of computational feasibility and generalizability, however. That is, the computational demands imposed by an increase in model complexity, due to the incorporation of more, and more detailed information of individual‐level behaviours, can quickly exceed the capabilities of modern‐day personal computers and require either very long run‐times or the implementation on high‐performance computer clusters. Furthermore, the limited availability of fine‐scaled data necessary to parameterize these models often restricts their use to the investigation of particular aspects of dengue epidemiology and mostly in one specific setting. Results obtained from these studies, although highly informative for the particular research question, are therefore not easily transferable to other epidemiological or geographical settings. This then implies that for research questions of a more general nature a balance needs to be struck between a model's biological and ecological realism and computational feasibility.

In many cases, the computational overhead of agent‐based models arises not necessarily from individual infection events but rather from keeping track of host and vector demography and ecology, for example the processes related to birth, death and ageing. As these are largely independent events, they are amenable to code parallelization and can thus benefit from graphics processing unit (GPU) acceleration, which specializes in attaining high arithmetic throughputs. Speed‐ups of anywhere between 10 and 1000 times from a serialized central processing unit (CPU) code are theoretically feasible, although in practice this very much depends on the particular model and implementation. That is, the benefits of GPU acceleration can easily get lost on increasingly refined spatial resolutions, for example, when more time is spent keeping track of individuals' movements (which is generally not parallelizable) than updating infection statuses or age. Nevertheless, the gain in computational speed‐up through GPU acceleration enables us to simulate much bigger and more detailed models in significantly less time (see Box II).

The necessity to include higher‐resolution in biological scales such as population structure in modelling frameworks is clear, given the recent rise of ever more detailed genetic (Faria et al., 2017; Woolhouse et al., 2015), mobility (Kraemer, Perkins et al., 2015; Wesolowski et al., 2015; Lemey et al., 2014) or social/communication (Salathé, Bengtsson, Bodnar, Brewer, & Brownstein, 2012) data sets. That is, the increased difficulties (e.g., interpretation) arising from the use of more complex models were not compensated for by sufficient data to validate model output; however, this is now changing. Methodologies such as the one presented in Box II now offer major opportunities to test and create new hypotheses on the spread, evolution and control of communicable diseases.

The spatial resolution of the research question dictates the complexity level of community structuring required in a model. Nonspatial deterministic approaches provide a natural entry point in understanding the epidemiology of pathogens strictly across time (Nagao & Koelle, 2008; Rodriguez‐Barraquer, Mier‐y Teran‐Romero, Schwartz, Burke, & Cummings, 2014; Ferguson, Rodríguez‐Barraquer, Dorigatti, Mier‐y Teran‐Romero, & Laydon, 2016). Epidemiological questions directed over very small geographical areas, such as investigating the effects of vector distribution, or vector and host movement between an individual's home and workplace, require fine‐scale spatial models (Chao, Longini, & Halloran, 2013; Reiner, Stoddard, & Scott, 2014; Perkins, Paz‐Soldan, Stoddard, Morrison, & Forshey, 2016; Hladish, Pearson, Chao, Rojas, & Recchia, 2016). Coarser‐scaled network models are applied when individual movement between households becomes redundant, for example exploring the effects of national or international human movement, or environmental heterogeneities over large spatial regions, on the spread, evolution and control of communicable diseases.

In the context of DENV, such approaches are now reaching the capacity to be modulated and fitted to particular, existing host populations and to simulate real‐world scenarios in terms of transmission and control. This would be particularly valuable in the context of DENV vaccination modelling, which is recent in the literature (e.g., (Lourenço & Recker, 2016; Flasche et al., 2016; Chao et al., 2012; Coudeville & Garnett, 2012)). For most of the published studies, simplifying assumptions are made on population heterogeneities or age‐dependent factors, for instance. However, with the amelioration of computational costs, realistic vaccination campaigns can be assessed, including details related to relative targeting of rural versus urban settings or detailed catch‐up and routine strategies dependent on host age. In terms of vector control programmes, the actual location of water reservoirs, the influence of climate or host mobility networks can also be considered. In summary, these advanced computing techniques have the capacity to change our perspective of modelling frameworks from being based on groups, generally of individuals sharing similar properties or phenotypes, to a perspective of host‐pathogen systems focusing on the role of single individuals, and how the mechanics of transmission and evolution at that scale can emerge as self‐evident patterns at the level of the population.

## 3 CONCLUDING REMARKS

We have reviewed the biological and epidemiological background of dengue, together with the major achievements of computational approaches dedicated to the pathogen. In addition and in the context of its increasing success as a global threat to public health, we have also highlighted critical knowledge gaps and research underachievements that call for an urgent renewed focus and promotion of such methodologies. We argue that possible advancements, based on new processing strategies, real‐time data sources and modelling frameworks already implemented for other pathogens, are already at reach of the research community. These new approaches are expected to make a significant contribution to our understanding of the evolutionary ecology and immunology of the dengue virus and its control in the near future.

## CONFLICT OF INTEREST

The authors declare no conflict of interest.

## Appendix 1 Data Description

### 1.1

Brazilian data are for suspected case counts from the Sistema de Informação de Agravos de Notificação (national notifiable diseases information system, SINAN) database (Brazil SINAN, http://portalsinan.saude.gov.br/). The regions of north and south, as presented in Figure 1, present aggregated data (south: states of Paraná, Santa Catarina, Rio Grande do Sul; north: states of Rondônia, Acre, Amazonas, Roraima, Pará, Amapá, Tocantins).

Dengue incidence data for Puerto Rico were provided by Michael Johansson from the CDC Dengue Branch, comprising clinically suspected cases of dengue fever (DF) and dengue haemorrhagic fever (DHF) in Puerto Rico between 1986 and 2012. Serotype‐specific testing has varied over time, so we adjusted the serotype‐specific incidence data proportionally to match the incidence of all suspected cases by month. A shorter time series had previously been published and analysed in (Johansson, Cummings, & Glass, 2009).

Annual incidence of DHF in Thailand 1973–2009 is of clinically confirmed cases and was obtained from the annual epidemiological surveillance reports published by the Ministry of Public Health (website: epid.moph.go.th). Dengue serotype prevalence for Thailand 1973–1999 was based on children hospitalized at the Queen Sirikit National Institute of Child Health in Bangkok, as previously published in (Nisalak et al., 2003).

Yearly counts of dengue publications (1970–2016) were collected from PubMed under two lists (queries). The first contained the total number of publications per year dedicated to dengue, including all hits with reference to dengue in the title and abstract (counts collected using the “results by year” functionality in PubMed). The second was a subset of the first, which included the use of dynamic models in the context of dengue research (manually curated). Queries were performed on the 1st of February 2017.

## References

[eva12554-bib-0001] Adams, B. , & Boots, M. (2006). Modelling the relationship between antibody‐dependent enhancement and immunological distance with application to dengue. Journal of Theoretical Biology, 242, 337–346. 16631802 10.1016/j.jtbi.2006.03.002

[eva12554-bib-0002] Adams, B. , & Boots, M. (2010). How important is vertical transmission in mosquitoes for the persistence of dengue? Insights from a mathematical model. Epidemics, 2, 1–10. 21352772 10.1016/j.epidem.2010.01.001

[eva12554-bib-0003] Adams, B. , Holmes, E. C. , Zhang, C. , Mammen, M. P. , Nimmannitya, S. , Kalayanarooj, S. , & Boots, M. (2006). Crossprotective immunity can account for the alternating epidemic pattern of dengue virus serotypes circulating in Bangkok. Proceedings of the National Academy of Sciences of the United States of America, 103, 14234–14239. 16966609 10.1073/pnas.0602768103 PMC1599940

[eva12554-bib-0004] Adams, B. , & Kapan, D. D. (2009). Man bites mosquito: Understanding the contribution of human movement to vector‐borne disease dynamics. PLoS ONE, 4, e6763. 19707544 10.1371/journal.pone.0006763 PMC2727792

[eva12554-bib-0005] Aguiar, M. , Kooi, B. W. , & Stollenwerk, N. (2008). Epidemiology of dengue fever: A model with temporary cross‐immunity and possible secondary infection shows bifurcations and chaotic behaviour in wide parameter regions. Mathematical Modelling of Natural Phenomena, 3, 48–70.

[eva12554-bib-0006] Aiken, S. , & Leigh, C. (1978). Dengue haemorrhagic fever in South‐east Asia. Transactions of the Institute of British Geographers, 3, 476–497.

[eva12554-bib-0007] Alonso, D. , McKane, A. J. , & Pascual, M. (2007). Stochastic amplification in epidemics. Journal of the Royal Society Interface, 4, 575–582. 10.1098/rsif.2006.0192 PMC2373404 17251128

[eva12554-bib-0008] Amarilla, A. A. , de Almeida, F. T. , Jorge, D. M. , Alfonso, H. L. , de Castro‐Jorge, L. A. , Nogueira, N. A. , … Aquino, V. H. (2009). Genetic diversity of the E protein of dengue type 3 virus. Virology Journal, 6, 113. 19627608 10.1186/1743-422X-6-113 PMC2720943

[eva12554-bib-0009] Anders, K. L. , Nguyet, N. M. , Chau, N. V. V. , Hung, N. T. , Thuy, T. T. , Lien, L. B. , … Simmons, C. P. (2011). Epidemiological factors associated with dengue shock syndrome and mortality in hospitalized dengue patients in Ho Chi Minh City, Vietnam. American Journal of Tropical Medicine and Hygiene, 84, 127–134. 21212214 10.4269/ajtmh.2011.10-0476 PMC3005500

[eva12554-bib-0011] Anderson, K. B. , Gibbons, R. V. , Cummings, D. A. , Nisalak, A. , Green, S. , Libraty, D. H. , … Endy, T. P. (2014). A shorter time interval between first and second dengue infections is associated with protection from clinical illness in a school‐based cohort in Thailand. The Journal of Infectious Diseases, 209, 360–368. 23964110 10.1093/infdis/jit436 PMC3883164

[eva12554-bib-0012] Ansari, H. , & Hesaaraki, M. (2012). A with‐in host dengue infection model with immune response and Beddington‐DeAngelis incidence rate. Applied Mathematics, 03, 177–184.

[eva12554-bib-0013] Auguste, A. J. , Lemey, P. , Pybus, O. G. , Suchard, M. A. , Salas, R. A. , Adesiyun, A. A. , … Carrington, C. V. F. (2010). Yellow fever virus maintenance in Trinidad and its dispersal throughout the Americas. Journal of Virology, 84, 9967–9977. 20631128 10.1128/JVI.00588-10 PMC2937779

[eva12554-bib-0014] Aziz, A. T. , Al‐Shami, S. A. , Mahyoub, J. A. , Hatabbi, M. , Ahmad, A. H. , & Rawi, C. S. M. (2014). An update on the incidence of dengue gaining strength in Saudi Arabia and current control approaches for its vector mosquito. Parasites & Vectors, 7, 258. 24890567 10.1186/1756-3305-7-258 PMC4057576

[eva12554-bib-0015] Balmaseda, A. , Hammond, S. N. , Tellez, Y. , Imhoff, L. , Rodriguez, Y. , Saborío, S. I. , … Harris, E. (2006). High seroprevalence of antibodies against dengue virus in a prospective study of schoolchildren in Managua, Nicaragua. Tropical Medicine & International Health: TM & IH, 11, 935–942. 16772016 10.1111/j.1365-3156.2006.01641.x

[eva12554-bib-0016] Balsitis, S. J. , Williams, K. L. , Lachica, R. , Flores, D. , Kyle, J. L. , & Harris, E. (2010). Lethal antibody enhancement of dengue disease in mice is prevented by Fc modification. PLoS Pathogens, 6, e1000790. 20168989 10.1371/journal.ppat.1000790 PMC2820409

[eva12554-bib-0017] Bashyam, H. S. , Green, S. , & Rothman, A. L. (2006). Dengue virus‐reactive CD8+ T cells display quantitative and qualitative differences in their response to variant epitopes of heterologous viral serotypes. The Journal of Immunology, 176, 2817–2824. 16493038 10.4049/jimmunol.176.5.2817

[eva12554-bib-0018] Bedford, T. , Cobey, S. , & Pascual, M. (2011). Strength and tempo of selection revealed in viral gene genealogies. BMC Evolutionary Biology, 11, 220. 21787390 10.1186/1471-2148-11-220 PMC3199772

[eva12554-bib-0019] Bedford, T. , Suchard, M. A. , Lemey, P. , Dudas, G. , Gregory, V. , Hay, A. J. , … Rambaut, A. (2014). Integrating influenza antigenic dynamics with molecular evolution. eLife, 2014, 1–26. 10.7554/eLife.01914 PMC3909918 24497547

[eva12554-bib-0020] Beebe, N. W. , Cooper, R. D. , Mottram, P. , & Sweeney, A. W. (2009). Australia's dengue risk driven by human adaptation to climate change. PLoS Neglected Tropical Diseases, 3, e429. 19415109 10.1371/journal.pntd.0000429 PMC2671609

[eva12554-bib-0021] Beltramello, M. , Williams, K. L. , Simmons, C. P. , Macagno, A. , Simonelli, L. , Quyen, N. T. , … Sallusto, F. (2010). The human immune response to dengue virus is dominated by highly cross‐reactive antibodies endowed with neutralizing and enhancing activity. Cell Host & Microbe, 8, 271–283. 20833378 10.1016/j.chom.2010.08.007 PMC3884547

[eva12554-bib-0022] Bennett, S. N. , Drummond, A. J. , Kapan, D. D. , Suchard, M. A. , Muñoz‐Jordán, J. L. , Pybus, O. G. , … Gubler, D. J. (2010). Epidemic dynamics revealed in dengue evolution. Molecular Biology and Evolution, 27, 811–818. 19965886 10.1093/molbev/msp285 PMC2877535

[eva12554-bib-0023] Bennett, S. N. , Holmes, E. C. , Chirivella, M. , Rodriguez, D. M. , Beltran, M. , Vorndam, V. , … McMillan, W. O. (2003). Selection‐driven evolution of emergent dengue virus. Molecular Biology and Evolution, 20, 1650–1658. 12832629 10.1093/molbev/msg182

[eva12554-bib-0024] Bennett, S. N. , Holmes, E. C. , Chirivella, M. , Rodriguez, D. M. , Beltran, M. , Vorndam, V. , … McMillan, W. O. (2006). Molecular evolution of dengue 2 virus in Puerto Rico: Positive selection in the viral envelope accompanies clade reintroduction. Journal of General Virology, 87, 885–893. 16528038 10.1099/vir.0.81309-0

[eva12554-bib-0025] Ben‐Shachar, R. , & Koelle, K. (2014). Minimal within‐host dengue models highlight the specific roles of the immune response in primary and secondary dengue infections. Journal of the Royal Society Interface, 12, 20140886. https://doi.org/10.1098/rsif.2014.0886 10.1098/rsif.2014.0886 PMC4305404 25519990

[eva12554-bib-0026] Ben‐Shachar, R. , Schmidler, S. , & Koelle, K. (2016). Drivers of inter‐individual variation in dengue viral load dynamics. PLoS Computational Biology, 12, e1005194. 27855153 10.1371/journal.pcbi.1005194 PMC5113863

[eva12554-bib-0027] Bhoomiboonchoo, P. , Nisalak, A. , Chansatiporn, N. , Yoon, I. K. , Kalayanarooj, S. , Thipayamongkolgul, M. , … Gibbons, R. V. (2015). Sequential dengue virus infections detected in active and passive surveillance programs in Thailand, 1994–2010. BMC Public Health, 15, 250. 25886528 10.1186/s12889-015-1590-z PMC4371716

[eva12554-bib-0028] Bin Ghouth, A. S. , Amarasinghe, A. , & Letson, G. W. (2012). Dengue outbreak in Hadramout, Yemen, 2010: An epidemiological perspective. American Journal of Tropical Medicine and Hygiene, 86, 1072–1076. 22665621 10.4269/ajtmh.2012.11-0723 PMC3366525

[eva12554-bib-0029] Bosio, C. F. , Thomas, R. E. , Grimstad, P. R. , & Rai, K. S. (1992). Variation in the efficiency of vertical transmission of dengue‐1 virus by strains of *Aedes albopictus* (Diptera: Culicidae). Journal of Medical Entomology, 29, 985–989. 1460640 10.1093/jmedent/29.6.985

[eva12554-bib-0030] Brathwaite Dick, O. , San Martín, J. L. , Montoya, R. H. , del Diego, J. , Zambrano, B. , & Dayan, G. H. (2012). The history of dengue outbreaks in the Americas. American Journal of Tropical Medicine and Hygiene, 87, 584–593. 23042846 10.4269/ajtmh.2012.11-0770 PMC3516305

[eva12554-bib-0031] Brazil SINAN . Sistema de informacao de agravos de notificacao (national notifiable diseases information system) http://portalsinan.saude.gov.br/

[eva12554-bib-0032] Bull, J. J. , & Turelli, M. (2013). Wolbachia versus dengue: Evolutionary forecasts. Evolution, Medicine, and Public Health, 2013, 197–207. 10.1093/emph/eot018 PMC3847891 24481199

[eva12554-bib-0033] Burke, D. S. , Nisalak, A. , Johnson, D. E. , & Scott, R. M. (1988). A prospective study of dengue infections in Bangkok. American Journal of Tropical Medicine and Hygiene, 38, 172–180. 3341519 10.4269/ajtmh.1988.38.172

[eva12554-bib-0034] Calisher, C. H. , Karabatsos, N. , Dalrymple, J. M. , Shope, R. E. , Porterfield, J. S. , Westaway, E. G. , & Brandt, W. E. (1989). Antigenic relationships between flaviviruses as determined by cross‐neutralization tests with polyclonal antisera. Journal of General Virology, 70(Pt 1), 37–43. 2543738 10.1099/0022-1317-70-1-37

[eva12554-bib-0035] Campbell, K. M. , Haldeman, K. , Lehnig, C. , Munayco, C. V. , Halsey, E. S. , Laguna‐Torres, V. A. , … Scott, T. W. (2015). Weather regulates location, timing, and intensity of dengue virus transmission between humans and mosquitoes. PLoS Neglected Tropical Diseases, 9, e0003957. 26222979 10.1371/journal.pntd.0003957 PMC4519153

[eva12554-bib-0036] Carrington, C. V. F. , Foster, J. E. , Pybus, O. G. , Bennett, S. N. , & Holmes, E. C. (2005). Invasion and maintenance of dengue virus type 2 and type 4 in the Americas. Journal of Virology, 79, 14680–14687. 16282468 10.1128/JVI.79.23.14680-14687.2005 PMC1287558

[eva12554-bib-0037] Cavrini, F. , Gaibani, P. , Pierro, A. M. , Rossini, G. , Landini, M. P. , & Sambri, V. (2009). Chikungunya: An emerging and spreading arthropod‐borne viral disease. Journal of Infection in Developing Countries, 3, 744–752. 20009275 10.3855/jidc.169

[eva12554-bib-0038] Chao, D. L. , Halstead, S. B. , Halloran, M. E. , & Longini, I. M. (2012). Controlling dengue with vaccines in Thailand. PLoS Neglected Tropical Diseases, 6, e1876. 23145197 10.1371/journal.pntd.0001876 PMC3493390

[eva12554-bib-0039] Chao, D. L. , Longini, I. M. , & Halloran, M. E. (2013). The effects of vector movement and distribution in a mathematical model of dengue transmission. PLoS ONE, 8, e76044. 24204590 10.1371/journal.pone.0076044 PMC3804532

[eva12554-bib-0040] Chau, T. N. B. , Hieu, N. T. , Anders, K. L. , Wolbers, M. , Lien, L. B. , Hieu, L. T. , … Simmons, C. P. (2009). Dengue virus infections and maternal antibody decay in a prospective birth cohort study of Vietnamese infants. The Journal of Infectious Diseases, 200, 1893–1900. 19911991 10.1086/648407 PMC4340501

[eva12554-bib-0041] Chew, C. H. , Woon, Y. L. , Amin, F. , Adnan, T. H. , Abdul Wahab, A. H. , Ahmad, Z. E. , … Lim, T. O. (2016). Ruralurban comparisons of dengue seroprevalence in Malaysia. BMC Public Health, 16, 824. 27538986 10.1186/s12889-016-3496-9 PMC4991088

[eva12554-bib-0042] Choudhury, M. A. , Lott, W. B. , Banu, S. , Cheng, A. Y. , Teo, Y. Y. , Ong, R. T. , & Aaskov, J. (2015). Nature and extent of genetic diversity of dengue viruses determined by 454 pyrosequencing. PLoS ONE, 10, e0142473. 26566128 10.1371/journal.pone.0142473 PMC4643897

[eva12554-bib-0043] Clapham, H. E. , Quyen, T. H. , Kien, D. T. H. , Dorigatti, I. , Simmons, C. P. , Ferguson, N. M. (2016). Modelling virus and antibody dynamics during dengue virus infection suggests a role for antibody in virus clearance. PLoS Computational Biology, 12, e1004951. 27213681 10.1371/journal.pcbi.1004951 PMC4877086

[eva12554-bib-0044] Clapham, H. E. , Tricou, V. , N., Van Vinh Chau , Simmons, C. P. , & Ferguson, N. M. (2014). Within‐host viral dynamics of dengue serotype 1 infection. Journal of the Royal Society Interface, 11, 20140094. 10.1098/rsif.2014.0094 PMC4032531 24829280

[eva12554-bib-0045] Coudeville, L. , & Garnett, G. P. (2012). Transmission dynamics of the four dengue serotypes in southern Vietnam and the potential impact of vaccination. PLoS ONE, 7, e51244. 23251466 10.1371/journal.pone.0051244 PMC3519629

[eva12554-bib-0046] Cummings, D. A. T. , Irizarry, R. A. , Huang, N. E. , Endy, T. P. , Nisalak, A. , Ungchusak, K. , & Burke, D. S. (2004). Travelling waves in the occurrence of dengue haemorrhagic fever in Thailand. Nature, 427, 344–347. 14737166 10.1038/nature02225

[eva12554-bib-0047] Cummings, D. A. T. , Schwartz, I. B. , Billings, L. , Shaw, L. B. , & Burke, D. S. (2005). Dynamic effects of antibody‐dependent enhancement on the fitness of viruses. Proceedings of the National Academy of Sciences of the United States of America, 102, 15259–15264. 16217017 10.1073/pnas.0507320102 PMC1257724

[eva12554-bib-0048] Cuong, H. Q. , Hien, N. T. , Duong, T. N. , Phong, T. V. , Cam, N. N. , Farrar, J. , … Horby, P. (2011). Quantifying the emergence of dengue in Hanoi, Vietnam: 1998–2009. PLoS Neglected Tropical Diseases, 5, e1322. 21980544 10.1371/journal.pntd.0001322 PMC3181236

[eva12554-bib-0049] Dejnirattisai, W. , Jumnainsong, A. , Onsirisakul, N. , Fitton, P. , Vasanawathana, S. , Limpitikul, W. , … Screaton, G. (2010). Cross‐reacting antibodies enhance dengue virus infection in humans. Science, 328, 745–748. 20448183 10.1126/science.1185181 PMC3837288

[eva12554-bib-0050] Dorigatti, I. , Aguas, R. , Donnelly, C. A. , Guy, B. , Coudeville, L. , Jackson, N. , … Ferguson, N. M. (2015). Modelling the immunological response to a tetravalent dengue vaccine from multiple phase‐2 trials in Latin America and South East Asia. Vaccine, 33, 3746–3751. 26051515 10.1016/j.vaccine.2015.05.059 PMC4504002

[eva12554-bib-0051] Duangchinda, T. , Dejnirattisai, W. , Vasanawathana, S. , Limpitikul, W. , Tangthawornchaikul, N. , Malasit, P. , … Screaton, G. (2010). Immunodominant T‐cell responses to dengue virus NS3 are associated with DHF. Proceedings of the National Academy of Sciences of the United States of America, 107, 16922–16927. 20837518 10.1073/pnas.1010867107 PMC2947904

[eva12554-bib-0052] Faria, N. R. , Lourenço, J. , Marques de Cerqueira, E. , Maia de Lima, M. , & Carlos Junior Alcantara, L. (2016). Epidemiology of Chikungunya virus in Bahia, Brazil, 2014–2015. PLoS Currents, https://doi.org/10.1371/currents.outbreaks.c97507e3e48efb946401755d468c28b2 10.1371/currents.outbreaks.c97507e3e48efb946401755d468c28b2 PMC4747681 27330849

[eva12554-bib-0053] Faria, N. R. , Quick, J. , Claro, I. M. , Thézé, J. , de Jesus, J. G. , Giovanetti, M. , … Pybus, O. G. (2017). Establishment andcryptic transmission of Zika virus in Brazil and the Americas. Nature, 546, 406–410. 28538727 10.1038/nature22401 PMC5722632

[eva12554-bib-0054] Faria, N. R. , Sabino, E. C. , Nunes, M. R. T. , Alcantara, L. C. J. , Loman, N. J. , & Pybus, O. G. (2016). Mobile real‐time surveillance of Zika virus in Brazil. Genome Medicine, 8, 97. 27683027 10.1186/s13073-016-0356-2 PMC5041528

[eva12554-bib-0055] Faria, N. R. , Suchard, M. A. , Rambaut, A. , Streicker, D. G. , & Lemey, P. (2013). Simultaneously reconstructing viral cross‐species transmission history and identifying the underlying constraints. Philosophical Transactions of the Royal Society of London Series B, Biological Sciences, 368, 20120196. 23382420 10.1098/rstb.2012.0196 PMC3678322

[eva12554-bib-0056] Ferguson, N. , Anderson, R. , & Gupta, S. (1999). The effect of antibody‐dependent enhancement on the transmission dynamics and persistence of multiple‐strain pathogens. Proceedings of the National Academy of Sciences of the United States of America, 96, 790–794. 9892712 10.1073/pnas.96.2.790 PMC15215

[eva12554-bib-0057] Ferguson, N. M. , Rodríguez‐Barraquer, I. , Dorigatti, I. , Mier‐y Teran‐Romero, L. , Laydon, D. J. , & Cummings, D. A. T. (2016). Benefits and risks of the Sanofi‐Pasteur dengue vaccine: Modeling optimal deployment. Science, 353, 1033–1036. 27701113 10.1126/science.aaf9590 PMC5268127

[eva12554-bib-0058] Fernandez‐Garcia, M. D. , Mazzon, M. , Jacobs, M. , & Amara, A. (2009). Pathogenesis of flavivirus infections: Using and abusing the host cell. Cell Host & Microbe, 5, 318–328. 19380111 10.1016/j.chom.2009.04.001

[eva12554-bib-0059] Filomatori, C. V. , Carballeda, J. M. , Villordo, S. M. , Aguirre, S. , Pallarés, H. M. , Maestre, A. M. , … Gamarnik, A. V. (2017). Dengue virus genomic variation associated with mosquito adaptation defines the pattern of viral non‐coding RNAs and fitness in human cells. PLOS Pathogens, 13, e1006265. 28264033 10.1371/journal.ppat.1006265 PMC5354447

[eva12554-bib-0060] Flasche, S. , Jit, M. , Rodríguez‐Barraquer, I. , Coudeville, L. , Recker, M. , Koelle, K. , … Ferguson, N. (2016). The long‐term safety, public health impact, and cost‐effectiveness of routine vaccination with a recombinant, live‐attenuated dengue vaccine (Dengvaxia): A model comparison study. PLoS Medicine, 13, e1002181. 27898668 10.1371/journal.pmed.1002181 PMC5127514

[eva12554-bib-0061] Forshey, B. M. , Reiner, R. C. , Olkowski, S. , Morrison, A. C. , Espinoza, A. , Long, K. C. , … Stoddard, S. T. (2016). Incomplete protection against dengue virus type 2 re‐infection in Peru. PLoS Neglected Tropical Diseases, 10, 1–17. 10.1371/journal.pntd.0004398 PMC4746126 26848841

[eva12554-bib-0062] Francis, T. (1960). On the doctrine of original antigenic sin. Proceedings of the American Philosophical Society, 104, 572–578.

[eva12554-bib-0063] Franco, L. , Di Caro, A. , Carletti, F. , Vapalahti, O. , Renaudat, C. , Zeller, H. , & Tenorio, A. (2010). Recent expansion of dengue virus serotype 3 inWest Africa. Euro Surveillance, 15, 2005–2008. 20184854

[eva12554-bib-0064] Gardy, J. , Loman, N. J. , & Rambaut, A. (2015). Real‐time digital pathogen surveillance the time is now. Genome Biology, 16, 155. 27391693 10.1186/s13059-015-0726-x PMC4531805

[eva12554-bib-0065] Gautret, P. , Botelho‐Nevers, E. , Charrel, R. N. , & Parola, P. (2010). Dengue virus infections in travellers returning from Benin to France, July‐August 2010. Euro Surveillance, 15, 2–3. 20843471

[eva12554-bib-0066] Gibbons, R. V. , Kalanarooj, S. , Jarman, R. G. , Nisalak, A. , Vaughn, D. W. , Endy, T. P. , … Srikiatkhachorn, A. (2007). Analysis of repeat hospital admissions for dengue to estimate the frequency of third or fourth dengue infections resulting in admissions and dengue hemorrhagic fever, and serotype sequences. American Journal of Tropical Medicine and Hygiene, 77, 910–913. 17984352

[eva12554-bib-0067] Gjenero‐Margan, I. , Aleraj, B. , Krajcar, D. , Lesnikar, V. , Klobučar, A. , Pem‐Novosel, I. , … Mlinarić‐Galinović, G. (2011). Autochthonous dengue fever in Croatia, August–September 2010. Euro Surveillance, 16, 19805. 21392489

[eva12554-bib-0068] Green, S. , Vaughn, D. W. , Kalayanarooj, S. , Nimmannitya, S. , Suntayakorn, S. , Nisalak, A. , … Ennis, F. A. (1999). Early immune activation in acute dengue illness is related to development of plasma leakage and disease severity. The Journal of Infectious Diseases, 179, 755–762. 10068569 10.1086/314680

[eva12554-bib-0069] Grenfell, B. T. , Pybus, O. G. , Gog, J. R. , Wood, J. L. N. , Daly, J. M. , Mumford, J. A. , & Holmes, E. C. (2004). Unifying the epidemiological and evolutionary dynamics of pathogens. Science (New York, NY), 303, 327–332. 10.1126/science.1090727 14726583

[eva12554-bib-0070] Gubler, D. J. (1998). Dengue and dengue hemorrhagic fever. Clinical Microbiology Reviews, 11, 480–496. 9665979 10.1128/cmr.11.3.480 PMC88892

[eva12554-bib-0071] Gujarati, T. P. , & Ambika, G. (2014). Virus antibody dynamics in primary and secondary dengue infections. Journal of Mathematical Biology, 69, 1773–1800. 24384697 10.1007/s00285-013-0749-4

[eva12554-bib-0072] Guzman, M. G. , Halstead, S. B. , Artsob, H. , Buchy, P. , Farrar, J. , Gubler, D. J. , … Peeling, R. W. (2010). Dengue: A continuing global threat. Nature Reviews Microbiology, 8, S7–S16. 21079655 10.1038/nrmicro2460 PMC4333201

[eva12554-bib-0073] Guzman, M. G. , & Kouri, G. (2008). Dengue haemorrhagic fever integral hypothesis: Confirming observations, 1987–2007. Transactions of the Royal Society of Tropical Medicine and Hygiene, 102, 522–523. 18420239 10.1016/j.trstmh.2008.03.001

[eva12554-bib-0074] Halstead, S. B. (2007). Dengue. Lancet, 370, 1644–1652. 17993365 10.1016/S0140-6736(07)61687-0

[eva12554-bib-0075] Halstead, S. B. (2011). Antibody, macrophages, dengue virus infection, shock, and hemorrhage: A pathogenetic cascade. Reviews of Infectious Diseases, 11(Suppl 4), S830–S839. 10.1093/clinids/11.supplement_4.s830 2665015

[eva12554-bib-0076] Halstead, S. B. , Mahalingam, S. , Marovich, M. A. , Ubol, S. , & Mosser, D. M. (2010). Intrinsic antibody‐dependent enhancement of microbial infection in macrophages: Disease regulation by immune complexes. The Lancet Infectious Diseases, 10, 712–722. 20883967 10.1016/S1473-3099(10)70166-3 PMC3057165

[eva12554-bib-0077] Halstead, S. B. , & O'Rourke, E. J. (1977). Dengue viruses and mononuclear phagocytes. I. Infection enhancement by non‐neutralizing antibody. The Journal of Experimental Medicine, 146, 201–217. 406347 10.1084/jem.146.1.201 PMC2180729

[eva12554-bib-0078] Halstead, S. B. , Rojanasuphot, S. , & Sangkawibha, N. (1983). Original antigenic sin in dengue. American Journal of Tropical Medicine and Hygiene, 32, 154–156. 6824120 10.4269/ajtmh.1983.32.154

[eva12554-bib-0079] Hang, V. T. T. , Holmes, E. C. , Duong, V. , Nguyen, T. Q. , Tran, T. H. , Quail, M. , … Simmons, C. P. (2010). Emergence of the Asian 1 genotype of dengue virus serotype 2 in Viet Nam: In vivo fitness advantage and lineage replacement in South‐East Asia. PLoS Neglected Tropical Diseases, 4, e757. 20651932 10.1371/journal.pntd.0000757 PMC2907417

[eva12554-bib-0080] Hladish, T. J. , Pearson, C. A. B. , Chao, D. L. , Rojas, D. P. , Recchia, G. L. , Gómez‐Dantés, H. , … Longini, I. M. (2016). Projected impact of dengue vaccination in Yucatán, Mexico. PLoS Neglected Tropical Diseases, 10, 1–19. 10.1371/journal.pntd.0004661 PMC4882069 27227883

[eva12554-bib-0082] Holmes, E. C. (2003). Patterns of intra‐ and interhost nonsynonymous variation reveal strong purifying selection in dengue virus. Journal of Virology, 77, 11296–11298. 14512579 10.1128/JVI.77.20.11296-11298.2003 PMC224983

[eva12554-bib-0083] Holmes, E. C. (2008). Evolutionary history and phylogeography of human viruses. Annual Review of Microbiology, 62, 307–328. 10.1146/annurev.micro.62.081307.162912 18785840

[eva12554-bib-0084] Holmes, E. C. , & Twiddy, S. S. (2003). The origin, emergence and evolutionary genetics of dengue virus. Infection, Genetics and Evolution, 3, 19–28. 10.1016/s1567-1348(03)00004-2 12797969

[eva12554-bib-0085] Houldcroft, C. J. , Beale, M. A. , & Breuer, J. (2017). Clinical and biological insights from viral genome sequencing. Nature Reviews Microbiology, 15, 183–192. 28090077 10.1038/nrmicro.2016.182 PMC7097211

[eva12554-bib-0086] Hu, K. , Thoens, C. , Bianco, S. , Edlund, S. , Davis, M. , Douglas, J. , & Kaufman, J. H. (2013). The effect of antibodydependent enhancement, cross immunity, and vector population on the dynamics of dengue fever. Journal of Theoretical Biology, 319, 62–74. 23206388 10.1016/j.jtbi.2012.11.021

[eva12554-bib-0087] Imrie, A. , Meeks, J. , Gurary, A. , Sukhbataar, M. , Kitsutani, P. , Effler, P. , & Zhao, Z. (2007). Differential functional avidity of dengue virus‐specific T‐cell clones for variant peptides representing heterologous and previously encountered serotypes. Journal of Virology, 81, 10081–10091. 17626101 10.1128/JVI.00330-07 PMC2045385

[eva12554-bib-0088] Irie, K. , Mohan, P. M. , Sasaguri, Y. , Putnak, R. , & Padmanabhan, R. (1989). Sequence analysis of cloned dengue virus type 2 genome (New Guinea‐C strain). Gene, 75, 197–211. 2714651 10.1016/0378-1119(89)90266-7

[eva12554-bib-0089] Issack, M. I. (2010). Reemergence of dengue in Mauritius. Emerging Infectious Diseases, 16, 3–5. 10.3201/eid1604.091582 PMC3321960 20350397

[eva12554-bib-0090] Jaenisch, T. , Junghanss, T. , Wills, B. , Brady, O. J. , Eckerle, I. , Farlow, A. , & Sankoh, O. (2014). Dengue expansion in Africa‐Not recognized or not happening? Emerging Infectious Diseases, 20. 10.3201/eid2010.140487 PMC4193177 25271370

[eva12554-bib-0091] Jain, A. , & Chaturvedi, U. C. (2010). Dengue in infants: An overview. FEMS Immunology and Medical Microbiology, 59, 119–130. 20402771 10.1111/j.1574-695X.2010.00670.x PMC7110389

[eva12554-bib-0092] Johansson, M. A. , Cummings, D. A. T. , & Glass, G. E. (2009). Multiyear climate variability and dengue‐El Nino southern oscillation, weather, and dengue incidence in Puerto Rico, Mexico, and Thailand: A longitudinal data analysis. PLoS Medicine, 6, e1000168. 19918363 10.1371/journal.pmed.1000168 PMC2771282

[eva12554-bib-0093] Johansson, M. A. , Dominici, F. , & Glass, G. E. (2009). Local and global effects of climate on dengue transmission in Puerto Rico. PLoS Neglected Tropical Diseases, 3, e382. 19221592 10.1371/journal.pntd.0000382 PMC2637540

[eva12554-bib-0094] Karl, S. , Halder, N. , Kelso, J. K. , Ritchie, S. A. , & Milne, G. J. (2014). A spatial simulation model for dengue virus infection in urban areas. BMC Infectious Diseases, 14, 447. 25139524 10.1186/1471-2334-14-447 PMC4152583

[eva12554-bib-0095] Katzelnick, L. C. , Coloma, J. , & Harris, E. (2017). Dengue: Knowledge gaps, unmet needs, and research priorities. The Lancet Infectious Diseases, 17, e88–e100. 28185868 10.1016/S1473-3099(16)30473-X PMC5967882

[eva12554-bib-0096] Ke, R. , Aaskov, J. , Holmes, E. C. , & Lloyd‐Smith, J. O. (2013). Phylodynamic analysis of the emergence and epidemiological impact of transmissible defective dengue viruses. PLoS Pathogens, 9, e1003193. 23468631 10.1371/journal.ppat.1003193 PMC3585136

[eva12554-bib-0097] Khan, J. , Khan, I. , & Amin, I. (2016). A comprehensive entomological, serological and molecular study of 2013 dengue outbreak of Swat, Khyber Pakhtunkhwa, Pakistan. PLoS ONE, 11, e0147416. 26848847 10.1371/journal.pone.0147416 PMC4746065

[eva12554-bib-0098] Klungthong, C. , Zhang, C. , Mammen, M. P. , Ubol, S. , & Holmes, E. C. (2004). The molecular epidemiology of dengue virus serotype 4 in Bangkok, Thailand. Virology, 329, 168–179. 15476884 10.1016/j.virol.2004.08.003

[eva12554-bib-0099] Kochel, T. J. , Watts, D. M. , Gozalo, A. S. , Ewing, D. F. , Porter, K. R. , & Russell, K. L. (2005). Cross‐serotype neutralization of dengue virus in *Aotus nancymae* monkeys. The Journal of Infectious Diseases, 191, 1000–1004. 15717278 10.1086/427511

[eva12554-bib-0100] Kochel, T. J. , Watts, D. M. , Halstead, S. B. , Hayes, C. G. , Espinoza, A. , Felices, V. , … Russell, K. L. (2002). Effect of dengue‐1 antibodies on American dengue‐2 viral infection and dengue haemorrhagic fever. Lancet, 360, 310–312. 12147378 10.1016/S0140-6736(02)09522-3

[eva12554-bib-0101] Kraemer, M. U. G. , Perkins, T. A. , Cummings, D. A. T. , Zakar, R. , Hay, S. I. , Smith, D. L. , & Reiner, R. C. (2015). Big city, small world: Density, contact rates, and transmission of dengue across Pakistan. Journal of the Royal Society Interface, 12, 20150468. 10.1098/rsif.2015.0468 PMC4614486 26468065

[eva12554-bib-0102] Kraemer, M. U. G. , Sinka, M. E. , Duda, K. A. , Mylne, A. Q. N. , Shearer, F. M. , Barker, C. M. , … Hay, S. I. (2015). The global distribution of the arbovirus vectors *Aedes aegypti* and *Ae. albopictus* . eLife, 4, 1–18. 10.7554/eLife.08347 PMC4493616 26126267

[eva12554-bib-0103] Kuno, G. , Chang, G. J. , Tsuchiya, K. R. , Karabatsos, N. , & Cropp, C. B. (1998). Phylogeny of the genus Flavivirus. Journal of Virology, 72, 73–83. 9420202 10.1128/jvi.72.1.73-83.1998 PMC109351

[eva12554-bib-0104] Kyle, J. L. , & Harris, E. (2008). Global spread and persistence of dengue. Annual Review of Microbiology, 62, 71–92. 10.1146/annurev.micro.62.081307.163005 18429680

[eva12554-bib-0105] Larrieu, S. , Dehecq, J. S. , Balleydier, E. , Jaffar, M. C. , Michault, A. , Vilain, P. , … Filleul, L. (2012). Re‐emergence of dengue in Reunion, France, January to April 2012. Euro Surveillance, 17, 20173. 22642944

[eva12554-bib-0106] Leitmeyer, K. C. , Vaughn, D. W. , Watts, D. M. , Salas, R. , Chacon, I. V. D. E. , Ramos, C. , & Rico‐Hesse, R. (1999). Dengue virus structural differences that correlate with pathogenesis. Journal of Virology, 73, 4738–4747. 10233934 10.1128/jvi.73.6.4738-4747.1999 PMC112516

[eva12554-bib-0107] Lemey, P. , Rambaut, A. , Bedford, T. , Faria, N. , Bielejec, F. , Baele, G. , … Suchard, M. A. (2014). Unifying viral genetics and human transportation data to predict the global transmission dynamics of human influenza H3N2. PLoS Pathogens, 10, e1003932. 24586153 10.1371/journal.ppat.1003932 PMC3930559

[eva12554-bib-0108] Lequime, S. , Fontaine, A. , Ar Gouilh, M. , Moltini‐Conclois, I. , & Lambrechts, L. (2016). Genetic drift, purifying selection and vector genotype shape dengue virus intrahost genetic diversity in mosquitoes. PLOS Genetics, 12, e1006111. 27304978 10.1371/journal.pgen.1006111 PMC4909269

[eva12554-bib-0109] Lourenco, J. , Maia de Lima, M. , Faria, N. R. , Walker, A. , Kraemer, M. U. G. , Villabona‐Arenas, C. J. , … Recker, M. (2017). Epidemiological and ecological determinants of Zika virus transmission in an urban setting. eLife, 6, e29820. 28887877 10.7554/eLife.29820 PMC5638629

[eva12554-bib-0110] Lourenço, J. , & Recker, M. (2013). Natural, persistent oscillations in a spatial multi‐strain disease system with application to dengue. PLoS Computational Biology, 9, e1003308. 24204241 10.1371/journal.pcbi.1003308 PMC3812071

[eva12554-bib-0111] Lourenço, J. , & Recker, M. (2010). Viral and epidemiological determinants of the invasion dynamics of novel dengue genotypes. PLoS Neglected Tropical Diseases, 4, e894. 21124880 10.1371/journal.pntd.0000894 PMC2990689

[eva12554-bib-0112] Lourenço, J. , & Recker, M. (2014). The 2012 Madeira dengue outbreak: Epidemiological determinants and future epidemic potential. PLoS Neglected Tropical Diseases, 8, e3083. 25144749 10.1371/journal.pntd.0003083 PMC4140668

[eva12554-bib-0113] Lourenço, J. , & Recker, M. (2016). Dengue serotype immune‐interactions and their consequences for vaccine impact predictions. Epidemics, 16, 40–48. 27663790 10.1016/j.epidem.2016.05.003 PMC5030310

[eva12554-bib-0114] Magnus, C. (2013). Virus neutralisation: New insights from kinetic neutralisation curves. PLoS Computational Biology, 9, e1002900. 23468602 10.1371/journal.pcbi.1002900 PMC3585397

[eva12554-bib-0115] Mangada, M. M. , Endy, T. P. , Nisalak, A. , Chunsuttiwat, S. , Vaughn, D. W. , Libraty, D. H. , … Rothman, A. L. (2002). Dengue‐specific T cell responses in peripheral blood mononuclear cells obtained prior to secondary dengue virus infections in Thai schoolchildren. The Journal of Infectious Diseases, 185, 1697–1703. 12085313 10.1086/340822

[eva12554-bib-0117] Martin, E. , Chirivella, M. , Co, J. K. G. , Santiago, G. A. , Gubler, D. J. , Muñoz‐Jordán, J. L. , & Bennett, S. N. (2016). Insights into the molecular evolution of dengue virus type 4 in Puerto Rico over two decades of emergence. Virus Research, 213, 23–31. 26569594 10.1016/j.virusres.2015.11.009 PMC4767545

[eva12554-bib-0118] Minayev, P. , & Ferguson, N. (2009). Incorporating demographic stochasticity into multistrain epidemic models: Application to influenza A. Journal of the Royal Society Interface, 6, 989–996. 10.1098/rsif.2008.0467 PMC2827439 19158010

[eva12554-bib-0119] Miralles, R. , Gerrish, P. J. , Moya, A. , & Elena, S. F. (1999). Clonal interference and the evolution of RNA viruses. Science, 285, 1745–1747. 10481012 10.1126/science.285.5434.1745

[eva12554-bib-0120] Mongkolsapaya, J. , Dejnirattisai, W. , Xiao‐ning, X. , Vasanawathana, S. , Tangthawornchaikul, N. , Chairunsri, A. , … Screaton, G. (2003). Original antigenic sin and apoptosis in the pathogenesis of dengue hemorrhagic fever. Nature Medicine, 9, 921–927. 10.1038/nm887 12808447

[eva12554-bib-0121] Montoya, M. , Gresh, L. , Mercado, J. C. , Williams, K. L. , Vargas, M. J. , Gutierrez, G. , … Harris, E. (2013). Symptomatic versus inapparent outcome in repeat dengue virus infections is influenced by the time interval between infections and study year. PLoS Neglected Tropical Diseases, 7, e2357. 23951377 10.1371/journal.pntd.0002357 PMC3738476

[eva12554-bib-0122] Morrison, A. C. , Minnick, S. L. , Rocha, C. , Forshey, B. M. , Stoddard, S. T. , Getis, A. , … Kochel, T. J. (2010). Epidemiology of dengue virus in Iquitos, Peru 1999 to 2005: Interepidemic and epidemic patterns of transmission. PLoS Neglected Tropical Diseases, 4, e670. 20454609 10.1371/journal.pntd.0000670 PMC2864256

[eva12554-bib-0123] Murray, N. E. A. , Quam, M. B. , & Wilder‐Smith, A. (2013). Epidemiology of dengue: Past, present and future prospects. Clinical Epidemiology, 5, 299–309. 23990732 10.2147/CLEP.S34440 PMC3753061

[eva12554-bib-0124] Nagao, Y. , & Koelle, K. (2008). Decreases in dengue transmission may act to increase the incidence of dengue hemorrhagic fever. Proceedings of the National Academy of Sciences of the United States of America, 105, 2238–2243. 18250338 10.1073/pnas.0709029105 PMC2538904

[eva12554-bib-0125] Nagao, Y. , Svasti, P. , Tawatsin, A. , & Thavara, U. (2008). Geographical structure of dengue transmission and its determinants in Thailand. Epidemiology and Infection, 136, 843–851. 17624231 10.1017/S0950268807008990 PMC2870862

[eva12554-bib-0126] Nikin‐Beers, R. , & Ciupe, S. M. (2015). The role of antibody in enhancing dengue virus infection. Mathematical Biosciences, 263, 83–92. 25707916 10.1016/j.mbs.2015.02.004

[eva12554-bib-0127] Nisalak, A. , Endy, T. P. , Nimmannitya, S. , Kalayanarooj, S. , Thisayakorn, U. , Scott, R. M. , … Vaughn, D. W. (2003). Serotype‐specific dengue virus circulation and dengue disease in Bangkok, Thailand from 1973 to 1999. American Journal of Tropical Medicine and Hygiene, 68, 191–202. 12641411

[eva12554-bib-0128] Nunes, M. R. T. , Faria, N. R. , de Vasconcelos, J. M. , Golding, N. , Kraemer, M. U. G. , de Oliveira, L. F. , … Vasconcelos, P. F. (2015). Emergence and potential for spread of Chikungunya virus in Brazil. BMC Medicine, 13, 102. 25976325 10.1186/s12916-015-0348-x PMC4433093

[eva12554-bib-0129] Nunes, M. R. T. , Faria, N. R. , Vasconcelos, H. B. , de Almeida Medeiros, D. B. , de Lima, C. P. S. , Carvalho, V. L. , … Vasconcelos, P. F. (2012). Phylogeography of dengue virus serotype 4, Brazil, 2010–2011. Emerging Infectious Diseases, 18, 1858–1864. 23092706 10.3201/eid1811.120217 PMC3559147

[eva12554-bib-0130] Nunes, M. R. T. , Palacios, G. , Faria, N. R. , Sousa, E. C. , Pantoja, J. A. , Rodrigues, S. G. , … Lipkin, W. I. (2014). Air travel is associated with intracontinental spread of dengue virus serotypes 1–3 in Brazil. PLoS Neglected Tropical Diseases, 8, e2769. 24743730 10.1371/journal.pntd.0002769 PMC3990485

[eva12554-bib-0131] Nuraini, N. , Tasman, H. , Soewono, E. , & Sidarto, K. A. (2009). A with‐in host dengue infection model with immune response. Mathematical and Computer Modelling, 49, 1148–1155.

[eva12554-bib-0132] OhAinle, M. , Balmaseda, A. , Macalalad, A. R. , Tellez, Y. , Zody, M. C. , Saborío, S. , … Harris, E. (2011). Dynamics of dengue disease severity determined by the interplay between viral genetics and serotype‐specific immunity. Science Translational Medicine, 3, 114ra128. 10.1126/scitranslmed.3003084 PMC4517192 22190239

[eva12554-bib-0133] Olkowski, S. , Forshey, B. M. , Morrison, A. C. , Rocha, C. , Vilcarromero, S. , Halsey, E. S. , … Stoddard, S. T. (2013). Reduced risk of disease during postsecondary dengue virus infections. The Journal of Infectious Diseases, 208, 1026–1033. 23776195 10.1093/infdis/jit273 PMC3749012

[eva12554-bib-0134] Parreira, R. , Centeno‐Lima, S. , Lopes, A. , Portugal‐Calisto, D. , Constantino, A. , & Nina, J. (2014). Dengue virus serotype 4 and Chikungunya virus coinfection in a traveller returning from Luanda, Angola, January 2014. Euro Surveillance, 19, 6–9. 24650864 10.2807/1560-7917.es2014.19.10.20730

[eva12554-bib-0135] Pérez‐Sautu, U. , Costafreda, M. I. , Caylà, J. , Tortajada, C. , Lite, J. , Bosch, A. , & Pintó, R. M. (2011). Hepatitis A virus vaccine escape variants and potential new serotype emergence. Emerging Infectious Diseases, 17, 734–737. 21470474 10.3201/eid1704.101169 PMC3377408

[eva12554-bib-0136] Perkins, T. A. , Garcia, A. J. , Paz‐Soldán, V. A. , Stoddard, S. T. , Reiner, R. C. , Vazquez‐Prokopec, G. , … Tatem, A. J. (2014). Theory and data for simulating fine‐scale human movement in an urban environment. Journal of the Royal Society Interface, 11, 20140642. 10.1098/rsif.2014.0642 PMC4233749 25142528

[eva12554-bib-0137] Perkins, T. A. , Paz‐Soldan, V. A. , Stoddard, S. T. , Morrison, A. C. , Forshey, B. M. , Long, K. C. , … Prokopec, G. M. (2016). Calling in sick: Impacts of fever on intra‐urban human mobility. Proceedings of the Royal Society of London B: Biological Sciences, 283, 36–51. 10.1098/rspb.2016.0390 PMC4947886 27412286

[eva12554-bib-0138] Poblap, T. , Nitatpattana, N. , Chaimarin, A. , Barbazan, P. , Chauvancy, G. , Yoksan, S. , & Gonzalez, J. P. (2006). Silent transmission of virus during a Dengue epidemic, Nakhon Pathom Province, Thailand 2001. The Southeast Asian Journal of Tropical Medicine and Public Health, 37, 899–903. 17333731

[eva12554-bib-0139] Radke, E. G. , Gregory, C. J. , Kintziger, K. W. , Sauber‐Schatz, E. K. , Hunsperger, E. A. , Gallagher, G. R. , … Blackmore, C. G. M. (2012). Dengue outbreak in Key West, Florida, USA, 2009. Emerging Infectious Diseases, 18, 135–137. 22257471 10.3201/eid1801.110130 PMC3310087

[eva12554-bib-0140] Rasmussen, D. A. , Boni, M. F. , & Koelle, K. (2014). Reconciling phylodynamics with epidemiology: The case of dengue virus in southern Vietnam. Molecular Biology and Evolution, 31, 258–271. 24150038 10.1093/molbev/mst203 PMC3907054

[eva12554-bib-0141] Read, A. F. , Baigent, S. J. , Powers, C. , Kgosana, L. B. , Blackwell, L. , Smith, L. P. , … Nair, V. K. (2015). Imperfect vaccination can enhance the transmission of highly virulent pathogens. PLoS Biology, 13, 1–18. 10.1371/journal.pbio.1002198 PMC4516275 26214839

[eva12554-bib-0142] Recker, M. , Blyuss, K. B. , Simmons, C. P. , Hien, T. T. , Wills, B. , Farrar, J. , & Gupta, S. (2009). Immunological serotype interactions and their effect on the epidemiological pattern of dengue. Proceedings of the Royal Society of London B: Biological Sciences, 276, 2541–2548. 10.1098/rspb.2009.0331 PMC2684681 19369266

[eva12554-bib-0143] Reich, N. G. , Shrestha, S. , King, A. A. , Rohani, P. , Lessler, J. , Kalayanarooj, S. , … Cummings, D. A. (2013). Interactions between serotypes of dengue highlight epidemiological impact of cross‐immunity. Journal of the Royal Society Interface, 10, 20130414. 10.1098/rsif.2013.0414 PMC3730691 23825116

[eva12554-bib-0144] Reiner, R. C. , Stoddard, S. T. , & Scott, T. W. (2014). Socially structured human movement shapes dengue transmission despite the diffusive effect of mosquito dispersal. Epidemics, 6, 30–36. 24593919 10.1016/j.epidem.2013.12.003 PMC3971836

[eva12554-bib-0145] Rodriguez‐Barraquer, I. , Mier‐y Teran‐Romero, L. , Schwartz, I. B. , Burke, D. S. , & Cummings, D. A. T. (2014). Potential opportunities and perils of imperfect dengue vaccines. Vaccine, 32, 514–520. 24269318 10.1016/j.vaccine.2013.11.020 PMC4142437

[eva12554-bib-0146] Roth, A. , Mercier, A. , Lepers, C. , Hoy, D. , Duituturaga, S. , Benyon, E. , … Souares, Y. (2014). Concurrent outbreaks of dengue, chikungunya and Zika virus infections – An unprecedented epidemic wave of mosquito‐borne viruses in the Pacific 2012–2014. Euro Surveillance, 19, 1–8. 10.2807/1560-7917.es2014.19.41.20929 25345518

[eva12554-bib-0147] Rothman, A. L. (2011). Immunity to dengue virus: A tale of original antigenic sin and tropical cytokine storms. Nature Reviews Immunology, 11, 532–543. 10.1038/nri3014 21760609

[eva12554-bib-0148] Rothman, A. , & Ennis, F. (1999). Immunopathogenesis of dengue hemorrhagic fever. Virology, 6, 1–6. 10.1006/viro.1999.9656 10208914

[eva12554-bib-0149] Sabin, A. B. (1952). Research on dengue during World War II. American Journal of Tropical Medicine and Hygiene, 1, 30–50. 14903434 10.4269/ajtmh.1952.1.30

[eva12554-bib-0150] Salathé, M. , Bengtsson, L. , Bodnar, T. J. , Brewer, D. D. , Brownstein, J. S. , Buckee, C. , … Vespignani, A. (2012). Digital epidemiology. PLoS Computational Biology, 8, 1–5. 22844241 10.1371/journal.pcbi.1002616 PMC3406005

[eva12554-bib-0151] Salje, H. , Lessler, J. , Endy, T. P. , Curriero, F. C. , Gibbons, R. V. , Nisalak, A. , … Cummings, D. A. (2012). Revealing the microscale spatial signature of dengue transmission and immunity in an urban population. Proceedings of the National Academy of Sciences of the United States of America, 109, 9535–9538. 22645364 10.1073/pnas.1120621109 PMC3386131

[eva12554-bib-0152] San Martín, J. L. , Brathwaite, O. , Zambrano, B. , Solórzano, J. O. , Bouckenooghe, A. , Dayan, G. H. , & Guzmán, M. G. (2010). The epidemiology of dengue in the Americas over the last three decades: A worrisome reality. American Journal of Tropical Medicine and Hygiene, 82, 128–135. 20065008 10.4269/ajtmh.2010.09-0346 PMC2803522

[eva12554-bib-0153] Sangkawibha, N. , Rojanasuphot, S. , Ahandrik, S. , Viriyapongse, S. , Jatanasen, S. , Salitul, V. , … Halstead, S. B. (1984). Risk factors in dengue shock syndrome: A prospective epidemiologic study in Rayong, Thailand. I. The 1980 outbreak. American Journal of Epidemiology, 120, 653–669. 6496446 10.1093/oxfordjournals.aje.a113932

[eva12554-bib-0154] Schmitz, J. , Roehrig, J. , Barrett, A. , & Hombach, J. (2011). Next generation dengue vaccines: A review of candidates in preclinical development. Vaccine, 29, 7276–7284. 21781998 10.1016/j.vaccine.2011.07.017

[eva12554-bib-0155] Schwartz, L. M. , Halloran, M. E. , Durbin, A. P. , & Longini, I. M. J. (2015). The dengue vaccine pipeline: Implications for the future of dengue control. Vaccine, 33, 3293–3298. 25989449 10.1016/j.vaccine.2015.05.010 PMC4470297

[eva12554-bib-0156] Schwartz, I. B. , Shaw, L. B. , Cummings, D. A. T. , Billings, L. , McCrary, M. , & Burke, D. S. (2005). Chaotic desynchronization of multistrain diseases. Physical Review E, Statistical, Nonlinear, and Soft Matter Physics, 72, 66201. 10.1103/PhysRevE.72.066201 16486034

[eva12554-bib-0157] Schwarz, N. G. , Girmann, M. , Randriamampionona, N. , Bialonski, A. , Maus, D. , Krefis, A. C. , … Rakotozandrindrainy, R. (2012). Seroprevalence of antibodies against Chikungunya, Dengue, and Rift Valley fever viruses after febrile illness outbreak, Madagascar. Emerging Infectious Diseases, 18, 1780–1786. 23092548 10.3201/eid1811.111036 PMC3559170

[eva12554-bib-0158] Scott, T. W. , Morrison, A. C. , Lorenz, L. H. , Clark, G. G. , Strickman, D. , Kittayapong, P. , … Edman, J. D. (2000). Longitudinal studies of *Aedes aegypti* (Diptera: Culicidae) in Thailand and Puerto Rico: Population dynamics. Journal of Medical Entomology, 37, 77–88. 15218910 10.1603/0022-2585-37.1.77

[eva12554-bib-0159] Seidahmed, O. M. E. , Hassan, S. A. , Soghaier, M. A. , Siam, H. A. M. , Ahmed, F. T. A. , Elkarsany, M. M. , & Sulaiman, S. M. (2012). Spatial and temporal patterns of dengue transmission along a Red Sea coastline: A longitudinal entomological and serological survey in Port Sudan city. PLoS Neglected Tropical Diseases, 6, e1821. 23029582 10.1371/journal.pntd.0001821 PMC3459851

[eva12554-bib-0160] Shankarappa, R. (1999). Consistent viral evolutionary changes associated with the progression of human immunodeficiency virus type 1 infection. Journal of Virology, 73, 10489–10502. 10559367 10.1128/jvi.73.12.10489-10502.1999 PMC113104

[eva12554-bib-0161] Sierra, B. , García, G. , Pérez, A. B. , Morier, L. , Rodríguez, R. , Alvarez, M. , & Guzmán, M. G. (2002). Long‐term memory cellular immune response to dengue virus after a natural primary infection. International Journal of Infectious Diseases, 6, 125–128. 12121600 10.1016/s1201-9712(02)90073-1

[eva12554-bib-0162] Silva, M. M. O. , Rodrigues, M. S. , Paploski, I. A. D. , Kikuti, M. , Kasper, A. M. , Cruz, J. S. , … Ribeiro, G. S. (2016). Accuracy of dengue reporting by national surveillance system, Brazil. Emerging Infectious Diseases, 22, 336–339. 26812472 10.3201/eid2202.150495 PMC4734515

[eva12554-bib-0163] Sim, S. , Aw, P. P. K. , Wilm, A. , Teoh, G. , Hue, K. D. T. , Nguyen, N. M. , … Hibberd, M. L. (2015). Tracking dengue virus intra‐host genetic diversity during human‐to‐mosquito transmission. PLOS Neglected Tropical Diseases, 9, e0004052. 26325059 10.1371/journal.pntd.0004052 PMC4556672

[eva12554-bib-0164] Stephens, H. A. F. , Klaythong, R. , Sirikong, M. , Vaughn, D. W. , Green, S. , Kalayanarooj, S. , … Chandanayingyong, D. (2002). HLA‐A and ‐B allele associations with secondary dengue virus infections correlate with disease severity and the infecting viral serotype in ethnic Thais. Tissue Antigens, 60, 309–318. 12472660 10.1034/j.1399-0039.2002.600405.x

[eva12554-bib-0165] Tang, Y. , Rodpradit, P. , Chinnawirotpisan, P. , Mammen, M. P. J. , Li, T. , Lynch, J. A. , … Zhang, C. (2010). Comparative analysis of full‐length genomic sequences of 10 dengue serotype 1 viruses associated with different genotypes, epidemics, and disease severity isolated in Thailand over 22 years. American Journal of Tropical Medicine and Hygiene, 83, 1156–1165. 21036855 10.4269/ajtmh.2010.10-0052 PMC2963987

[eva12554-bib-0166] Tatem, A. J. , Hay, S. I. , & Rogers, D. J. (2006). Global traffic and disease vector dispersal. Proceedings of the National Academy of Sciences of the United States of America, 103, 6242–6247. 16606847 10.1073/pnas.0508391103 PMC1435368

[eva12554-bib-0167] Temporão, J. G. , Penna, G. O. , Carmo, E. H. , Coelho, G. E. , Azevedo, R. S. S. , Nunes, M. R. T. , & Vasconcelos, P. F. C. (2011). Dengue virus serotype 4, Roraima State, Brazil. Emerging Infectious Diseases, 17, 938–940. 21529421 10.3201/eid1705.101681 PMC3321786

[eva12554-bib-0168] Thai, K. T. D. , Cazelles, B. , Nguyen, N. V. , Vo, L. T. , Boni, M. F. , Farrar, J. , … de Vries, P. J. (2010). Dengue dynamics in Binh Thuan province, southern Vietnam: Periodicity, synchronicity and climate variability. PLoS Neglected Tropical Diseases, 4, e747. 20644621 10.1371/journal.pntd.0000747 PMC2903474

[eva12554-bib-0169] Thai, K. T. D. , Henn, M. R. , Zody, M. C. , Tricou, V. , Nguyet, N. M. , Charlebois, P. , … Simmons, C. P. (2012). High‐resolution analysis of intrahost genetic diversity in dengue virus serotype 1 infection identifies mixed infections. Journal of Virology, 86, 835–843. 22090119 10.1128/JVI.05985-11 PMC3255838

[eva12554-bib-0170] Thein, S. , Aung, M. M. , Shwe, T. N. , Aye, M. , Zaw, A. , Aye, K. , … Aaskov, J. (1997). Risk factors in dengue shock syndrome. American Journal of Tropical Medicine and Hygiene, 56, 566–572. 9180609 10.4269/ajtmh.1997.56.566

[eva12554-bib-0171] Thomas, S. J. , & Endy, T. P. (2011). Vaccines for the prevention of dengue: Development update. Human Vaccines, 7, 674–684. 21508679 10.4161/hv.7.6.14985

[eva12554-bib-0172] Thomas, D. L. , Santiago, G. A. , Abeyta, R. , Hinojosa, S. , Torres‐Velasquez, B. , Adam, J. K. , … Sharp, T. M. (2016). Reemergence of dengue in southern Texas, 2013. Emerging Infectious Diseases, 22, 1002–1007. 27191223 10.3201/eid2206.152000 PMC4880107

[eva12554-bib-0173] Thu, H. M. , Lowry, K. , Jiang, L. , Hlaing, T. , Holmes, E. C. , & Aaskov, J. (2005). Lineage extinction and replacement in dengue type 1 virus populations are due to stochastic events rather than to natural selection. Virology, 336, 163–172. 15892958 10.1016/j.virol.2005.03.018

[eva12554-bib-0174] Tsuzuki, A. , Vu, T. D. , Higa, Y. , Nguyen, T. Y. , & Takagi, M. (2009). High potential risk of dengue transmission during the hot‐dry season in Nha Trang City, Vietnam. Acta Tropica, 111, 325–329. 19467217 10.1016/j.actatropica.2009.05.010

[eva12554-bib-0175] Twiddy, S. S. , Farrar, J. J. , Vinh Chau, N. , Wills, B. , Gould, E. A. , Gritsun, T. , … Holmes, E. C. (2002). Phylogenetic relationships and differential selection pressures among genotypes of dengue‐2 virus. Virology, 298, 63–72. 12093174 10.1006/viro.2002.1447

[eva12554-bib-0176] Twiddy, S. S. , Holmes, E. C. , & Rambaut, A. (2003). Inferring the rate and time‐scale of dengue virus evolution. Molecular Biology and Evolution, 20, 122–129. 12519914 10.1093/molbev/msg010

[eva12554-bib-0177] Ubol, S. , & Halstead, S. B. (2010). How innate immune mechanisms contribute to antibody‐enhanced viral infections. Clinical and Vaccine Immunology, 17, 1829–1835. 20876821 10.1128/CVI.00316-10 PMC3008185

[eva12554-bib-0178] Vasilakis, N. , Cardosa, J. , Hanley, K. A. , Holmes, E. C. , & Weaver, S. C. (2012). Fever from the forest: Prospects for the continued emergence of sylvatic dengue virus and its impact on public health. Nature Reviews. Microbiology, 9, 532–541. 10.1038/nrmicro2595 PMC3321645 21666708

[eva12554-bib-0179] Vasilakis, N. , Holmes, E. C. , Fokam, E. B. , Faye, O. , Diallo, M. , Sall, A. A. , & Weaver, S. C. (2007). Evolutionary processes among sylvatic dengue type 2 viruses. Journal of Virology, 81, 9591–9595. 17553878 10.1128/JVI.02776-06 PMC1951459

[eva12554-bib-0116] Vasilakis, N. , & Weaver, S. C. (2008). The history and evolution of human dengue emergence. Advances in Virus Research, 72, 1–76. 19081488 10.1016/S0065-3527(08)00401-6

[eva12554-bib-0180] Volz, E. M. , Koelle, K. , & Bedford, T. (2013). Viral phylodynamics. PLoS Computational Biology, 9, e1002947. 23555203 10.1371/journal.pcbi.1002947 PMC3605911

[eva12554-bib-0181] Waggoner, J. J. , Balmaseda, A. , Gresh, L. , Sahoo, M. K. , Montoya, M. , Wang, C. , … Harris, E. (2016). Homotypic dengue virus reinfections in Nicaraguan children. The Journal of Infectious Diseases, 214, 986–993. 26984144 10.1093/infdis/jiw099 PMC5021223

[eva12554-bib-0182] Waman, V. P. , Kolekar, P. , Ramtirthkar, M. R. , Kale, M. M. , & Kulkarni‐Kale, U. (2016). Analysis of genotype diversity and evolution of Dengue virus serotype 2 using complete genomes. PeerJ, 4, e2326. 27635316 10.7717/peerj.2326 PMC5012332

[eva12554-bib-0183] Wearing, H. J. , & Rohani, P. (2006). Ecological and immunological determinants of dengue epidemics. Proceedings of the National Academy of Sciences of the United States of America, 103, 11802–11807. 16868086 10.1073/pnas.0602960103 PMC1544250

[eva12554-bib-0184] Weaver, S. C. , & Vasilakis, N. (2009). Molecular evolution of dengue viruses: Contributions of phylogenetics to understanding the history and epidemiology of the preeminent arboviral disease. Infection, Genetics and Evolution, 9, 523–540. 10.1016/j.meegid.2009.02.003 PMC3609037 19460319

[eva12554-bib-0185] Wesolowski, A. , Qureshi, T. , Boni, M. F. , Sundsøy, P. R. , Johansson, M. A. , Rasheed, S. B. , … Buckee, C. O. (2015). Impact of human mobility on the emergence of dengue epidemics in Pakistan. Proceedings of the National Academy of Sciences of the United States of America, 112, 11887–11892. 26351662 10.1073/pnas.1504964112 PMC4586847

[eva12554-bib-0186] Whitehorn, J. , & Simmons, C. P. (2011). The pathogenesis of dengue. Vaccine, 29, 7221–7228. 21781999 10.1016/j.vaccine.2011.07.022

[eva12554-bib-0187] Wichmann, O. , Yoon, I. K. , Vong, S. , Limkittikul, K. , Gibbons, R. V. , Mammen, M. P. , … Sabchareon, A. (2011). Dengue in Thailand and Cambodia: An assessment of the degree of underrecognized disease burden based on reported cases. PLoS Neglected Tropical Diseases, 5, e996. 21468308 10.1371/journal.pntd.0000996 PMC3066139

[eva12554-bib-0188] Williams, C. R. , Bader, C. A. , Kearney, M. R. , Ritchie, S. A. , & Russell, R. C. (2010). The extinction of dengue through natural vulnerability of its vectors. PLoS Neglected Tropical Diseases, 4, e922. 21200424 10.1371/journal.pntd.0000922 PMC3006136

[eva12554-bib-0189] Woolhouse, M. E. J. , Rambaut, A. , & Kellam, P. (2015). Lessons from Ebola: Improving infectious disease surveillance to inform outbreak management. Science Translational Medicine, 7, 307rv5. 10.1126/scitranslmed.aab0191 PMC5819730 26424572

[eva12554-bib-0190] Wrammert, J. , Onlamoon, N. , Akondy, R. S. , Perng, G. C. , Polsrila, K. , Chandele, A. , … Ahmed, R. (2012). Rapid and massive virus specific plasmablast responses during acute dengue virus infection in humans. Journal of Virology, 86, 2911–2918. 22238318 10.1128/JVI.06075-11 PMC3302324

[eva12554-bib-0191] Zhang, C. , Mammen, M. Jr , Chinnawirotpisan, P. , Klungthong, C. , Rodpradit, P. , Monkongdee, P. , … Holmes, E. C. (2005). Clade replacements in dengue virus serotypes 1 and 3 are associated with changing serotype prevalence. Journal of Virology, 79, 15123. 16306584 10.1128/JVI.79.24.15123-15130.2005 PMC1316048

